# Interleukin-10 as Covid-19 biomarker targeting KSK and its analogues: Integrated network pharmacology

**DOI:** 10.1371/journal.pone.0282263

**Published:** 2023-03-29

**Authors:** Vidhya Rajalakshmi V., Akilandeswari Ramu, Jayaprakash Chinnappan, Palanivel Velmurugan, Rajiv Pathak, Rami Adel Pashameah, Atif Abdulwahab A. Oyouni, Osama M. Al-Amer, Mohammed I. Alasseiri, Abdullah Hamadi, Mansuor A. Alanazi, Thangavelu Sathiamoorthi

**Affiliations:** 1 Anthropology and Health Informatics Lab, Department of Bioinformatics, Bharathiar University, Coimbatore, Tamil Nadu, India; 2 Centre for Materials Engineering and Regenerative Medicine, Bharath Institute of Higher Education and Research, Selaiyur, Chennai, Tamilnadu, India; 3 Department of Biotechnology, Himalayan Whitehouse International College, Kathmandu, Nepal; 4 Department of Chemistry, Faculty of Applied Science, Umm Al-Qura University, Makkah, Saudi Arabia; 5 Department of Biology, Faculty of Sciences, University of Tabuk, Tabuk, Saudi Arabia; 6 Genome and Biotechnology Unit, Faculty of Sciences, University of Tabuk, Tabuk, Saudi Arabia; 7 Department of Medical Laboratory Technology, Faculty of Applied Medical Sciences, University of Tabuk, Tabuk, Saudi Arabia; 8 Department of Microbiology, Faculty of Science, Alagappa University, Karikudi, Tamilnadu, India; Alagappa University, INDIA

## Abstract

COVID-19 caused by the SARS-CoV-2 virus is widespread in all regions, and it disturbs host immune system functioning leading to extreme inflammatory reaction and hyperactivation of the immune response. Kabasura Kudineer (KSK) is preventive medicine against viral infections and a potent immune booster for inflammation-related diseases. We hypothesize that KSK and KSK similar plant compounds, might prevent or control the COVID-19 infection in the human body. 1,207 KSK and KSK similar compounds were listed and screened via the Swiss ADME tool and PAINS Remover; 303 compounds were filtered including active and similar drug compounds. The targets were retrieved from similar drugs of the active compounds of KSK. Finally, 573 genes were listed after several screening steps. Next, network analysis was performed to finalize the potential target gene: construction of protein-protein interaction of 573 genes using STRING, identifying top hub genes in Cytoscape plug-ins (MCODE and cytoHubba). These ten hub genes play a crucial role in the inflammatory response. Target-miRNA interaction was also constructed using the miRNet tool to interpret miRNAs of the target genes and their functions. Functional annotation was done via DAVID to gain a complete insight into the mechanism of the enriched pathways and other diseases related to the given target genes. In Molecular Docking analysis, IL10 attained top rank in Target-miRNA interaction and also the gene formed prominent exchanges with an excellent binding score (> = -8.0) against 19 compounds. Among them, Guggulsterone has an acute affinity score of -8.8 for IL10 and exhibits anti-inflammatory and immunomodulatory properties. Molecular Dynamics simulation study also performed for IL10 and the interacting ligand compounds using GROMACS. Finally, Guggulsterone will be recommended to enhance immunity against several inflammatory diseases, including COVID19.

## 1. Introduction

The current COVID-19 pandemic caused by SARS-CoV-2 coronavirus has spread worldwide. It is emerging as the most dangerous infection affecting people. The first case was detected in Wuhan city, Hubei province of China, and by January 2020, WHO acknowledged it as a worldwide health crisis for its rapid spreading [1]. COVID-19 belongs to the Human Betacoronavirus genera (along with SARS-CoV and MERS-CoV), and they have many similarities, including immune escaping tactics, yet additional mechanisms SARS-CoV-2 remain undetected. Four structural proteins are S (spike) protein, M (membrane) protein, E (envelop) protein and N (nucleocapsid) protein helps the virus in pathogenesis [2]. There are five variants of COVID-19 which infect the people: alpha, beta, gamma, delta and currently omicron. COVID-19 severely infects the respiratory tract by creating a contact with the host’s ACE2 protein, which leads to difficulty in breathing, loss of smell, loss of taste, headache, cough, sneezing, fever, diarrhea, runny nose, sore throat are typical symptoms. Patients with COVID-19 might also develop severe symptoms of diseases like acute respiratory distress syndrome (ARDS), pneumonia and multiple organ failure [3].

The immune system protects the host from various infections caused by viruses and other microorganisms and produces antibodies to kill pathogens. It is the best defense against pathogens [4]. SARS-CoV-2 can disturb the normal functioning of the host’s immune system, thus leading to weakened immunity against infection and unconstrained inflammatory responses [5]. The COVID-19 infection is bolstered by a hostile inflammatory reaction leading to the release of many pro-inflammatory cytokines known as *cytokine storm*. The host’s immune response becomes hyperactive in response to the COVID-19 infection, leading to extreme inflammatory reactions and an assembly of diseases [6, 7]. Elevated levels of IL2, IL1B, IL4, IL5, IL7, IL8 (CXCL8), IFNG (Interferon-gamma), IL10, and TNF-alpha were found by clinical laboratory experiments [8, 9]. However, pattern recognition receptors (PRR), such as *Toll-like receptors* (TLR) and *NOD-like receptors* (NLR), are involved in the inflammatory response against severe oxidative stress. These are the primary reasons for initiating inflammatory responses by persuading pro-inflammatory cytokine gene expression [10].

Kabasura Kudineer has been described as a capable medicinal concoction for curing several viral infections, currently used to prevent COVID-19 infection. Disease 15 herbal plants used in the KSK concoction (*Zingiber officinale*, *Piper longum*, *Syzygium aromaticum*, *Tragia involucrata*, *Anacyclus pyrethrum*, *Hygrophila auriculata*, *Terminalia chebula*, *Justicia adhatoda*, *Coleus aromaticus*, *Saussurea costus*, *Tinospora cordifolia*, *Clerodendrum serratum*, *Andrographis paniculata*, *Sida acuta and Cyperus rotundus*). They possess potent anti-inflammatory, anti-viral, anti-bacterial, anti-fungal, inhibitor, hepato-protective, anti-pyretic, anti-asthmatic and immunomodulatory properties for managing several types of infections, especially in curing respiratory illnesses related to COVID-19. KSK could also be a potential immune booster for inflammation-related diseases [10, 11].

This study attempts to identify the natural compound and target via various computational approaches. The listed compounds were screened based on Lipinski violations, PAINS alerts, Bioavailability score, Solubility class and Synthetic accessibility in the SwissADME tool. A total of 573 genes were listed in which false positives were removed. Network analyses were done to find the top hub genes using STRING and Cytoscape (MCODE and cytoHubba). Additionally, miRNAs of those hub genes were searched through miRNet, a web-based platform for functional identification. MicroRNAs play an essential role in manipulating gene expression [12]. Enrichment analysis is also more beneficial for finding the Gene Ontology, pathway and other diseases related to the target hub genes. A molecular docking study using AutoDock Vina was implemented by calculating the docking score to verify the interaction between compounds and targets. This analysis also detailed how these novel drugs exert their therapeutic activity. Compound-Target-Pathway Network was constructed in Cytoscape to highlight the potential mechanism of action of the compounds. Finally, MD simulation for IL10 with the ligand molecules Herkinorin, Guggulsterone and mulberrofuran W selected based on the molecular docking results.

## 2. Materials and methods

**Fig 1:**
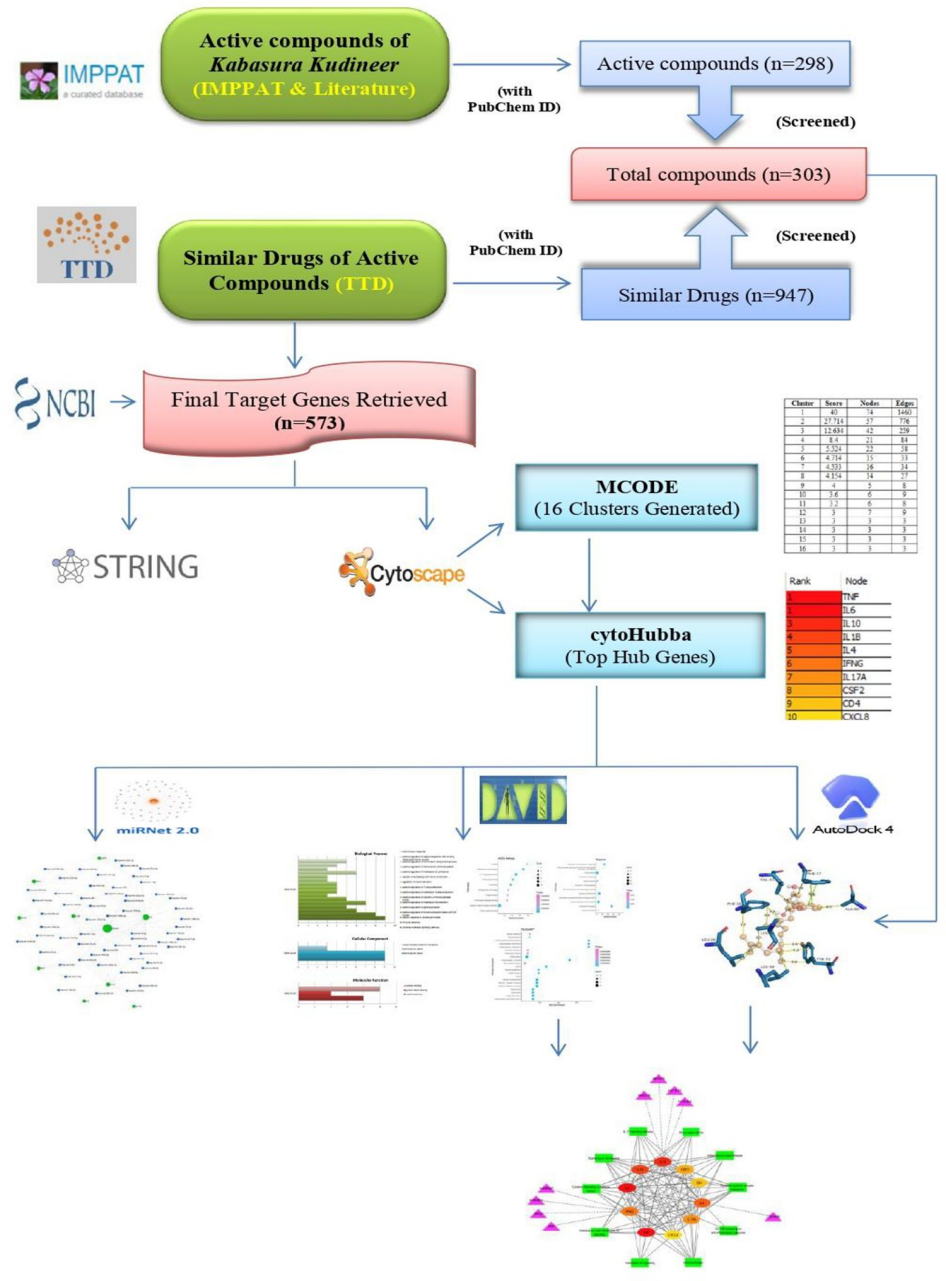
Flow chart of this research process.

Fig 1 displays the overall workflow of the present study.

### 2.1. Collection and screening of compounds

KSK concoction is composed of 15 plant ingredients. Phytoconstituents of these 15 plants were retrieved from literature reading and IMPPAT online database. IMPPAT is a technological platform for gathering information on traditional Indian medicinal plants and their phytochemical compounds [13]. Also, similar combinations of phytochemical compounds were recouped from TTD based on the high-similarity score of > = 85% [14, 15]. Duplicates, repeats, invalids, and no PubChem entry compounds were physically removed from the total compounds collection. Next, an important step is screening to identify the valid compounds based on the given criteria: Lipinski violations, PAINS alerts, Bioavailability score, Catechol A, Solubility class and Synthetic accessibility. PAINS Remover server and Swiss ADME tool were used for the screening process. Lastly, valid compounds were collected for further analysis [16].

### 2.2. Finding target genes

Target genes were retrieved in two ways: a). targets of similar compounds from TTD, and b). targets of COVID-19 from the NCBI Gene database (https://www.ncbi.nlm.nih.gov/gene). Both similar compound genes and COVID-19 genes were collected for network analysis and functional annotation.

### 2.3. Construction of protein-protein interaction network

The online STRING database was used for the construction of the PPI network. Listed target genes were uploaded, and *Homo sapiens* was nominated as a standard organism [17]. Nodes represent the genes, and edges represent the interaction between the nodes.

### 2.4. Cluster analysis and detecting hub genes

Cytoscape is a free, open-source platform to visualize complex networks, molecular interactions, and several plug-ins to perform diverse analyses [18]. MCODE is a Cytoscape plug-in to find highly interconnected nodes formed as clusters in a network. Top hub genes were identified in clusters. Hub genes were ranked via the MCC algorithm in cytoHubba [19].

### 2.5. MicroRNAs of the hub genes

miRNet is an online web-based tool to view the interaction of target genes-miRNA and interpret miRNAs’ functions. This tool works based on multiple-to-multiple relation; multiple genes can be regulated by one miRNA, and multiple miRNAs can regulate one gene [20]. miRNet is also used for exploring the functional annotation of genes; miRNA Family with their functions, diseases they are related to, associated clusters and transcription factors. A complex network has been simplified with the help of the Degree filter.

### 2.6. Key enrichment and pathway analyses

Functional Enrichment analysis was done via DAVID to understand the biological information of the genes [21]. GO terms: Biological Process, Cellular Components, Molecular Functions; Pathways Analysis: KEGG Pathway, Reactome; and Diseases related to the given target genes information were retrieved. Pathway and disease relation expression was shown in a bubble plot using ImageGP (http://www.ehbio.com/ImageGP/). GO enrichments results were established as a 2D bar graph using an MS Excel file.

### 2.7. Molecular docking analysis

The three-dimensional crystal structures for the target hub genes were downloaded from PDB [22]. The active sites of the protein structures were predicted by the CastP server (http://sts.bioe.uic.edu/). The missing residues in the target proteins were added using SwissPDB Viewer [23]. Water molecules were removed, and Kollman Charge hydrogen atoms were added for protein preparation in AutoDock [24, 25]. 3D structure of ligand molecules was downloaded from the PubChem database. Some ligands do not have a 3D structure in the PubChem database; their 2D files were downloaded and converted to the 3D structure using Chem Sketch [26]. MMFF94 force field parameter was used to optimize the compounds using OpenBabel [27] in Ubuntu. Molecular Docking analysis was done using the AutoDock Vina tool [28]. Protein-ligand 3D interactions were analyzed using PLIP software [29]. 3D and 2D hydrogen bond interactions of the protein-ligand complexes were visualized in PyMOL [30] (DeLano, 2002) and LigPlot+ [31]. The main aim of this molecular docking is to predict the interaction of the protein and ligand molecules and how they function as a complex. The result can be shown mainly based on the binding affinity of the protein-ligand complex. More negative the acute affinity score, the higher the critical relationship between the protein-ligand complexes.

### 2.8. Compound-target interaction network construction

After the Molecular Docking analysis was done, the interaction between the target proteins, ligand molecules, and significant pathways was predicted. This interaction was drawn as a Compound-Target-Pathway network in the Cytoscape tool. Network Analyzer is a Cytoscape plug-in used to compute the topological analysis for the constructed network. It gives information on the number of nodes and edges, neighbors, network diameter, radius, density, etc. Lastly, the calculated result was shown as summary table and node-specific tables [32].

### 2.9. Molecular dynamics simulation

The three dimensional protein structure of IL10 was downloaded from PDB and visualized in PyMOL. Molecular docking results for IL10 showed interactions with top four ligands: Herkinorin, (R,S)-homoaromaline hydrochloride, Guggulsterone and mulberrofuran W. Further MD simulation was performed for the protein-ligand complexes of IL10 and the above four ligands. MD simulation study was performed with GROMACS 2022.2 installed in Ubuntu on Windows. GROMACS is a software suite for molecular dynamics simulation [33]. The missing atoms were checked and filled using energy minimization with Spdb Viewer. Protein topology was created with ‘charmm36-jul2021’ force field. ‘pdb2gmx’ in GROMACS reads the.pdb file as input and select the specific force field depending upon the type of working system and add H atoms. It also generates GROMACS coordinate file and topology file. The force field needs to be compatible with the system. CGenFF online server was used for generating ligand topology files [34]. From both protein and ligand topologies,.gro files were combined for creating protein-ligand complex topology and saved as new file. Cubic grid box was generated and solvent system was added. This system was neutralized by adding SOD (positive) and CLA (negative). Energy minimization is a vital step to remove the steric clashes and improper geometries in the system. Steepest descent algorithm is used for the energy minimization. There are two different phases in equilibrating the system: (i) nvt–defines the constant number of particle volume and temperature (to stabilize the temperature); (ii) npt–defines the particle pressure and temperature of all constants. Final step is to run the simulation for the system using ‘mdrun’. The runtime for the simulation was 100ns for the system. There are major analyses to be done after MD simulation: (i) RMSD calculation–used to measure the scalar distance for the protein-ligand throughout the trajectory; (ii) RMSF calculation–used for characterizing local fluctuation in the model.

## 3. Results

### 3.1. Systematic extraction of active compounds

There are 321 active compounds present in the 15 plant ingredients of KSK. These 321 active compounds were collected from the literature and IMPPAT database. PubChem CIDs were retrieved for 1245 KSK compounds and KSK similar compounds [35] (S1 File). 1, 207 compounds were selected after removing the duplications. Table 1A clearly explains the selection criteria for compounds.

**Table 1:** Collection of compounds and target genes for the study. (a) shows the total count of KSK active compounds and total count of KSK similar compounds. There were 991 similar compounds in total in which 44 were removed for not having PubChem CIDs and 321 active compounds in total in which 23 were removed as they do not have PubChem CIDs. Totally 1,245 compounds were listed from which 38 duplicates were removed and 1,207 compounds were collected finally; (b) shows the number of target genes collected from TTD and NCBI databases.

	Description		Total Count
**(a)** **Number of Compounds collected**	KSK compounds (active compounds)		321
	KSK similar compounds		3799
	KSK Similar compounds—after duplication removed		991
	KSK Similar compounds—having PubChem CIDs		947
	KSK Active compounds -having PubChem CIDs		298
	**Total number of collective compounds (KSK and KSK similar)**		**1,207**
**(b)** **Number of target genes collected**	Targets collected from Similar Drug Compounds (**TTD**)	Total number of targets—TTD	1423
		After Duplicates removed	411
	Targets collected from **NCBI**	Total number of targets—NCBI	308
		After Duplicates removed	260
	Targets–as **STRING** Input	**Final count of Unique Targets after Invalid Genes removed for STRING Input**	**573**

Total compounds were screened to find the appropriate compounds to be taken for further steps. According to the GHS classification report, compounds with Catechol A property were removed because of their toxic nature [36] in the PAINS Remover server [37]. SwissADME was used to screen the compounds based on Lipinski’s violations (above 1 violation compounds excluded), PAINS alerts (0 alert compounds), Bioavailability score (0.55 minimum) and synthetic accessibility (1 to 7). Every drug molecule should obey the Lipinski rule and Bioavailability score [38]. Here, 303 compounds were obeyed out of 1,207 compounds (S2 File).

### 3.2. Target genes collection

False positives were removed and 573 unique genes were established from the whole genes (S3 File). A total number of collected target genes was highlighted in Table 1B.

### 3.3. Network analysis of target genes

**Fig 2:**
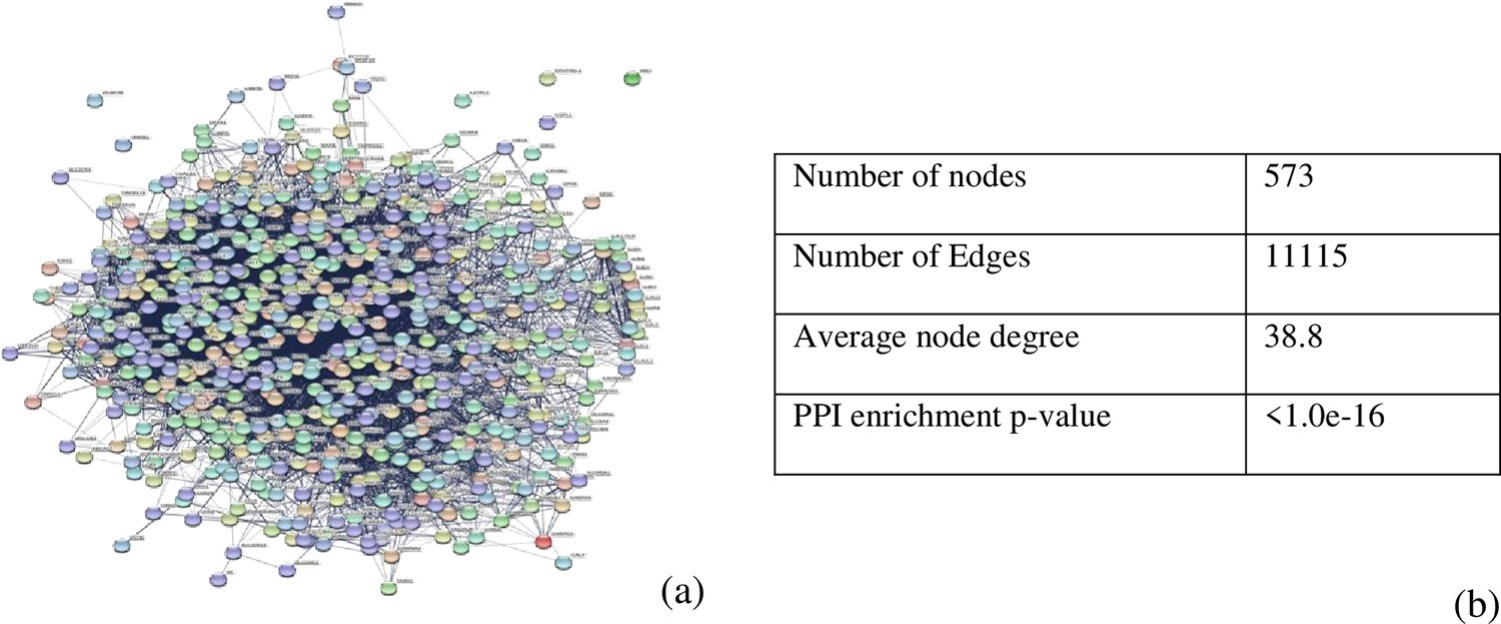
Construction of PPI network using STRING database. (a) PPI Network of Total Target Genes. (b) Network Stats of the obtained result.

The PPI network was constructed for the obtained target genes in the STRING database (Fig 2). Overall, 11,115 edges with 573 nodes interacted with each other. The constructed PPI network was visualized in Cytoscape 3.8.2 version. Cluster analysis was done in Cytoscape using the MCODE plug-in. 16 clusters were revealed from the STRING network (Table 2).

**Table 2:** MCODE result shows 16 clusters score, number of nodes and edges, and node IDs.

Cluster	Score	Nodes	Edges	Node IDs
1	40	74	1460	CSF1, NOS2, CXCL2, MMP3, IL9, ALB, IFNA1, PRF1, IL1R1, REN, CCL8, HAVCR2, IL4R, CXCL10, IL33, IL13, SELL, APOE, PPARG, CSF3, TLR3, CCL17, CD163, IL2, TLR7, IL6R, SERPINE1, IFNB1, TGFB1, MMP2, IL2RA, GPR29, EDN1, NOS3, IL2RG, CXCR3, CTLA4, IL7, IL22, IL17A, CAT, CCR1, FCGR1A, HMOX1, PTGS2, JAK2, HMGB1, CCL4, INS, NLRP3, IL18, FAS, HIF1A, CCR5, CD8A, CCL7, JUN, IL5, TLR2, SELE, ADIPOQ, FCGR3A, CCL3, NCAM1, STAT1, MMP1, SPP1, JAK1, GZMB, SOCS3, SOCS1, LEP, CCL20, PLG
2	27.714	57	776	HSPA4, CXCL8, CSF2, CCL27, THBS1, ACE, TLR4, APP, EGFR, KNG1, IL1B, STAT3, VWF, PDGFRB, CD209, SMAD3, FLT3, CRP, SIRT1, TNFRSF1B, CD4, IL4, IFIH1, SYK, IDO1, CCL2, ICAM1, MPO, AGTR1, KDR, SELP, MIF, LGALS3, RORC, IFNG, IFNAR2, LAG3, ELANE, IL10, GZMA, IL6, ESR1, MMP9, CASP8, AKT1, TNF, IFNA2, IL10RB, F3, AGT, CD14, LCN2, CD2, PPARA, MTOR, LY96, IL2RB
3	12.634	42	259	GJA5, F2, GJD4, MAF, GJC3, GJB5, HSPA5, ANGPT2, GJA10, GJA9, GJA4, SERPINC1, GJE1, THPO, GJB7, SERPINA1, PDGFRA, EPO, NFE2L2, GJA8, MMP8, GJA1, POMC, SDC1, GJD2, IGFBP3, GJB1, MCL1, GJA3, TEK, BECN1, SAA1, GJB2, GJD3, GJB4, IGF1R, GJB6, GJC1, GJC2, FLT1, VDR, GJB3
4	8.4	21	84	TRPM4, KCNH2, GABRG2, HTR1A, GABRB2, GABRA1, GABRA5, GRIN1, SLC1A1, HTR2A, CHRNA4, CACNA1A, GABRA3, KCNA3, HTR5A, GRM8, SLC6A1, GABRG3, SLC1A6, GRM2, SCN3A
5	5.524	22	58	TRPA1, GRM7, HTR1D, TRPM8, DRD2, HTR7, GABRA2, HTR2B, CACNA2D1, GRIK1, SLC1A2, SCN11A, GRIN2B, HRH1, SLC6A4, SCN5A, SLC6A3, SCN10A, CHRNB2, PRKCB, CYP2D6, KCNA5
6	4.714	15	33	TNNT2, COMT, TPH1, ADORA2B, MB, BCHE, MAOB, MAOA, XDH, SLC18A2, TPH2, TNNI3, ADORA1, AVP, ADORA2A
7	4.533	16	34	SREBF2, CTSD, FURIN, ATM, LIPE, CTSL, HNF4A, ANXA2, CDK6, FASN, SCD, NR1H3, NR1H4, HSPA8, HMGCR, TERT
8	4.154	14	27	FABP4, MBL2, PPARD, SERPING1, ADAMTS13, NR1I2, RXRA, F10, SERPIND1, DGAT1, APOH, SERPINF2, GC, LPA
9	4	5	8	AR, SP1, DNMT1, ESR2, CDK2
10	3.6	6	9	KIF11, TK1, TUBB, KIAA0101, TUBB4B, AURKB
11	3.2	6	8	HSD3B1, STS, AKR1C3, LSS, CYP1B1, SQLE
12	3	7	9	P2RX7, NOS1, CTSB, A2M, TRPV1, CALCA, CST3
13	3	3	3	ADM, GDF15, NPPB
14	3	3	3	HLA-G, HLA-B, HLA-C
15	3	3	3	ENPEP, AGTR2, PREP
16	3	3	3	SLC36A2, SLC36A1, SLC6A14

**Fig 3:**
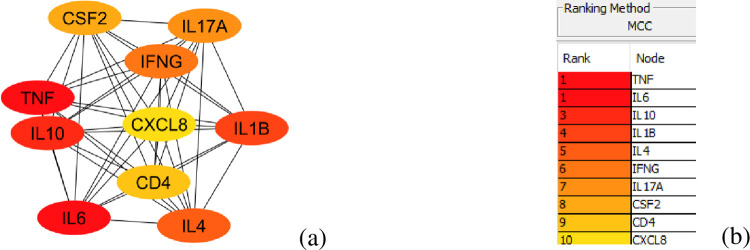
The final results are shown in cytoHubba. (a) PPI Network of the top 10 hub genes. The red color indicates the nodes with high MCC scores, and the yellow indicates the node with a low MCC score. (b) Ranking of the top 10 hub genes using the MCC algorithm.

After cluster generation, top hub genes were detected, showing highly interconnected regions. Hence, they were considered the significant nodes done by the cytoHubba plug-in in Cytoscape. TNF, IL6, IL10, IL1B, IL4, IFNG, IL17A, CSF2, CD4 and CXCL8 were the top 10 hub genes. These genes were ranked by the MCC method. MCC method is the most effective algorithm for finding high connectivity of nodes (Fig 3). Interestingly, these genes play an essential role in an extreme inflammatory reaction as the host immune response becomes hyperactive due to COVID-19 infection and it has been found by various clinical experiments.

**Fig 4:**
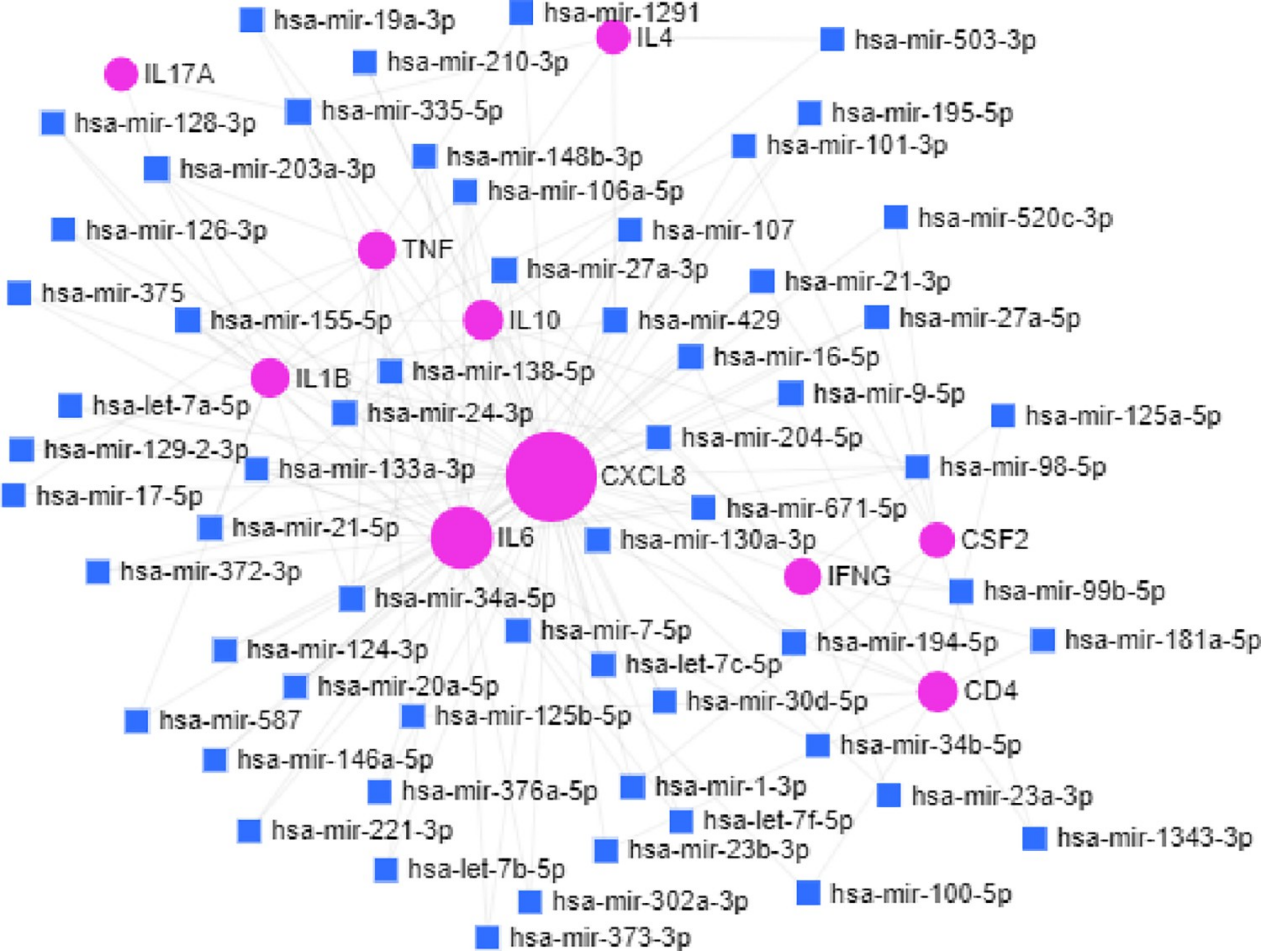
Target-miRNA interaction network constructed in miRNet online tool. Green nodes represent the target genes, and blue nodes represent miRNAs.

Furthermore, Target-miRNA interaction was constructed using an online tool called miRNet. This miRNet mainly focuses on interpreting the functions of the miRNAs through network-based analysis. The hub genes were given as a query list to build the network based on different miRNA databases integrated into miRNet. The results obtained from miRNet showed that 59 miRNAs interacted with 154 edges for the ten hub genes. The complex network was simplified using the Degree filter for reducing the least important nodes or edges with the Degree cut-off as 1.0 (as default), as shown in Fig 4. Table 3 provides the information on miRNAs’ interaction with genes.

**Table 3:** miRNAs of each hub genes given with the degree and the betweenness obtained from the miRNet tool.

Rank	Gene	Degree	Betweenness	miRNA
1	TNF	10	102.6344	hsa-mir-17-5p, hsa-mir-19a-3p, hsa-mir-24-3p, hsa-mir-34a-5p, hsa-mir-203a-3p, hsa-mir-125b-5p, hsa-mir-130a-3p, hsa-mir-155-5p, hsa-mir-7-5p, hsa-mir-1291
1	IL6	37	613.0374	hsa-let-7a-5p, hsa-let-7b-5p, hsa-let-7c-5p, hsa-let-7f-5p, hsa-mir-16-5p, hsa-mir-21-5p, hsa-mir-27a-3p, hsa-mir-98-5p, hsa-mir-106a-5p, hsa-mir-107, hsa-mir-34a-5p, hsa-mir-203a-3p, hsa-mir-1-3p, hsa-mir-124-3p, hsa-mir-9-5p, hsa-mir-146a-5p, hsa-mir-155-5p, hsa-mir-335-5p, hsa-mir-429, hsa-mir-100-5p, hsa-mir-128-3p, hsa-mir-375, hsa-mir-7-5p, hsa-mir-138-5p, hsa-mir-195-5p, hsa-mir-20a-5p, hsa-mir-21-3p, hsa-mir-221-3p, hsa-mir-27a-5p, hsa-mir-34b-5p, hsa-mir-376a-5p, hsa-mir-671-5p,hsa-mir-373-3p, hsa-mir-372-3p, hsa-mir-302a-3p, hsa-mir-148b-3p, hsa-mir-133a-3p
3	IL10	14	136.0619	hsa-let-7c-5p, hsa-mir-19a-3p, hsa-mir-21-5p, hsa-mir-27a-3p, hsa-mir-98-5p, hsa-mir-106a-5p, hsa-mir-107, hsa-mir-34a-5p, hsa-mir-194-5p, hsa-mir-155-5p, hsa-mir-503-3p, hsa-mir-671-5p, hsa-mir-210-3p, hsa-mir-30d-5p
4	IL1B	15	120.6849	hsa-mir-21-5p, hsa-mir-24-3p, hsa-mir-106a-5p, hsa-mir-34a-5p, hsa-mir-203a-3p, hsa-mir-204-5p, hsa-mir-155-5p, hsa-mir-429, hsa-mir-587, hsa-mir-101-3p, hsa-mir-126-3p, hsa-mir-128-3p, hsa-mir-129-2-3p, hsa-mir-375, hsa-mir-7-5p
5	IL4	4	19.64072	hsa-mir-24-3p, hsa-mir-335-5p, hsa-mir-429, hsa-mir-503-3p
6	IFNG	7	76.18816	hsa-mir-16-5p, hsa-mir-23a-3p, hsa-mir-24-3p, hsa-mir-27a-3p, hsa-mir-181a-5p, hsa-mir-125a-5p, hsa-mir-99b-5p
7	IL17A	2	0.6832723	hsa-mir-203a-3p, hsa-mir-335-5p
8	CSF2	7	43.2971	hsa-mir-1-3p, hsa-mir-1343-3p, hsa-mir-101-3p, hsa-mir-21-3p, hsa-mir-27a-5p, hsa-mir-34b-5p, hsa-mir-520c-3p
9	CD4	10	148.5669	hsa-mir-181a-5p, hsa-mir-204-5p, hsa-mir-125b-5p, hsa-mir-130a-3p, hsa-mir-9-5p, hsa-mir-125a-5p, hsa-mir-194-5p, hsa-mir-100-5p, hsa-mir-1343-3p, hsa-mir-23b-3p
10	CXCL8	48	1220.205	hsa-let-7a-5p, hsa-let-7b-5p, hsa-let-7c-5p, hsa-let-7f-5p, hsa-mir-16-5p, hsa-mir-17-5p, hsa-mir-21-5p, hsa-mir-23a-3p, hsa-mir-27a-3p, hsa-mir-98-5p, hsa-mir-106a-5p, hsa-mir-107, hsa-mir-34a-5p, hsa-mir-203a-3p, hsa-mir-204-5p, hsa-mir-1-3p, hsa-mir-124-3p, hsa-mir-130a-3p, hsa-mir-9-5p, hsa-mir-146a-5p, hsa-mir-194-5p, hsa-mir-155-5p, hsa-mir-335-5p, hsa-mir-429, hsa-mir-23b-3p, hsa-mir-99b-5p, hsa-mir-587, hsa-mir-101-3p, hsa-mir-126-3p, hsa-mir-129-2-3p, hsa-mir-7-5p, hsa-mir-138-5p, hsa-mir-195-5p, hsa-mir-20a-5p, hsa-mir-21-3p, hsa-mir-221-3p, hsa-mir-27a-5p, hsa-mir-376a-5p, hsa-mir-671-5p, hsa-mir-373-3p, hsa-mir-372-3p, hsa-mir-302a-3p, hsa-mir-148b-3p, hsa-mir-133a-3p, hsa-mir-210-3p, hsa-mir-30d-5p, hsa-mir-520c-3p, hsa-mir-1291

### 3.4. Functional annotation of the target genes

**Fig 5:**
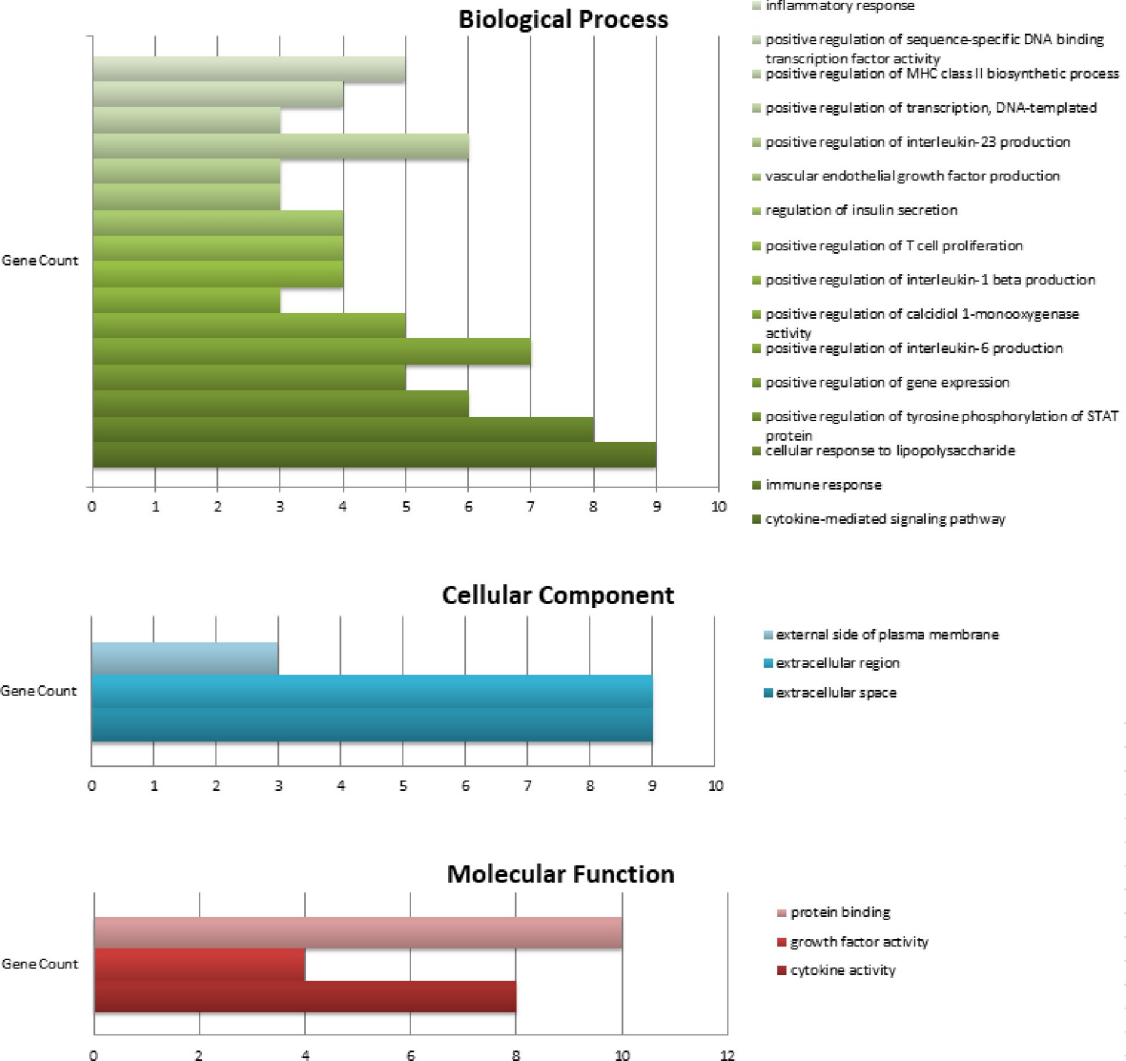
Gene Ontology Enrichment Analysis: Biological Process (BP), Cellular Component (CC) and Molecular Function (MF) were predicted for the given set of hub genes in the DAVID tool.

To gain further insight into the functions of target genes, functional annotation provides complete information on Gene Ontology Enrichment analysis (which includes Biological Processes, Molecular Functions and Cellular Components), Pathway analysis (KEGG pathway, Reactome) and Other Diseases (DisGeNET) that target genes are related with, and this can be achieved through DAVID. A cut-off criterion for a p-value of <0.05 was taken to indicate the significance. The top significantly enriched GO terms regarding Gene Ontology analysis are shown in Fig 5 and S4 File.

In Biological processes, cytokine-mediated signaling pathways and immune responses were the top significant enriched GO: BP terms. The cytokine-mediated signaling pathway involved nine genes IL10, IL4, IL6, CD4, CXCL8, CSF2, IL1B, TNF, and IL17A. Eight genes, IL10, IL4, CXCL8, CSF2, IFNG, IL1B, TNF and IL17A were involved in the immune response. In Cellular Component, nine genes IL10, IL4, IL6, CXCL8, CSF2, IFNG, IL1B, TNF and IL17A were found in the extracellular space and extracellular region. CD4, TNF and IL17A were located on the outer side of the plasma membrane. In Molecular Function, eight genes IL10, IL4, IL6, CSF2, IFNG, IL1B, TNF and IL17A, were found to have Cytokine activity. Four genes, IL10, IL4, IL6 and CSF2, were found to have growth factor activity.

**Fig 6:**
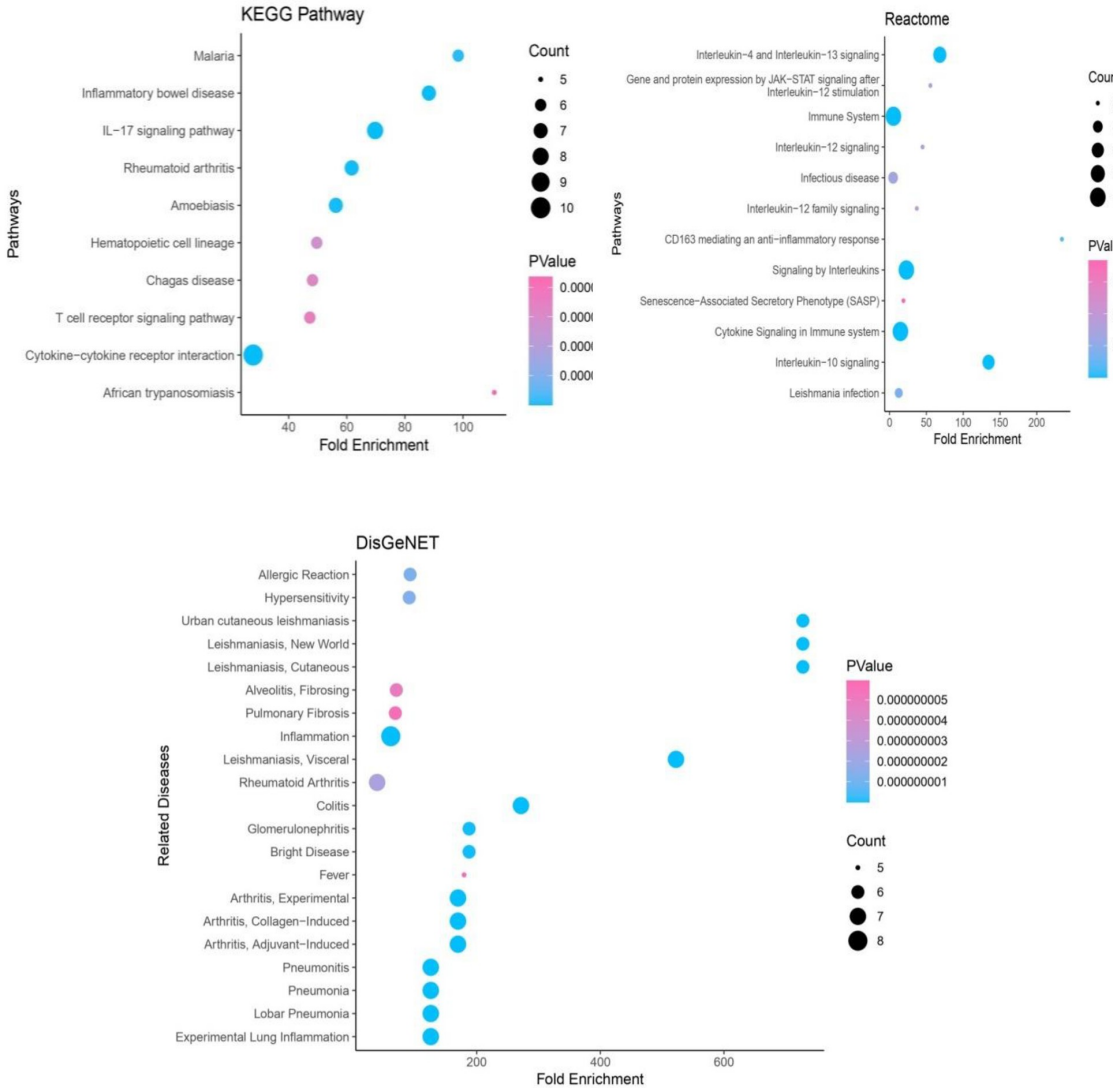
Bubble plot of the pathway enrichment analysis (KEGG and Reactome pathways) and DisGeNET (information on other diseases associated with the hub genes) of the resultant hub genes using DAVID. Fold Enrichment in the DAVID tool refers to the ratio of two proportions.

Ten hub genes were involved in cytokine-cytokine receptor interaction in the KEGG pathway. Eight genes, IL4, IL6, CXCL8, CSF2, IFNG, IL1B, TNF and IL17A were involved in the IL-17 signaling pathway. Seven genes IL10, IL4, IL6, IFNG, IL1B, TNF and IL17A, were engaged in Inflammatory bowel disease. In Reactome Pathway ten hub genes were involved in Signaling by Interleukins and Cytokine Signaling in the Immune system. Seven genes IL10, IL4, IL6, CXCL8, IL1B, TNF and IL17A were involved in Interleukin-4 and Interleukin-13 signaling. Six genes, IL10, IL6, CXCL8, CSF2, IL1B and TNF, were involved in Interleukin-10 signaling (Fig 6 and S4 File).

From the results of DisGeNET, the hub genes IL10, IL6, CXCL8, CSF2, IFNG, IL1B, and TNF were causing Visceral Leishmaniasis; IL10, IL4, CXCL8, IFNG, IL1B and TNF found to cause Cutaneous Leishmaniasis and Urban Cutaneous Leishmaniasis. Seven hub genes IL10, IL4, IL6, IFNG, IL1B, TNF and IL17A might be responsible for causing Colitis and Arthritis, except for Rheumatoid Arthritis. Seven hub genes, IL4, IL6, CSF2, IFNG, IL1B, TNF and IL17A, were responsible for Experimental Lung Inflammation, Pneumonitis, Pneumonia and Lobar Pneumonia. Eight genes IL10, IL6, CXCL8, CSF2, IFNG, IL1B, TNF and IL17A were involved in Inflammation. Interestingly, most of the diseases mentioned above have a direct or indirect link with the COVID-19 infection. Our results were discussed with the existing literature.

### 3.5. Analyzing protein-ligand interaction by molecular docking

**Fig 7:**
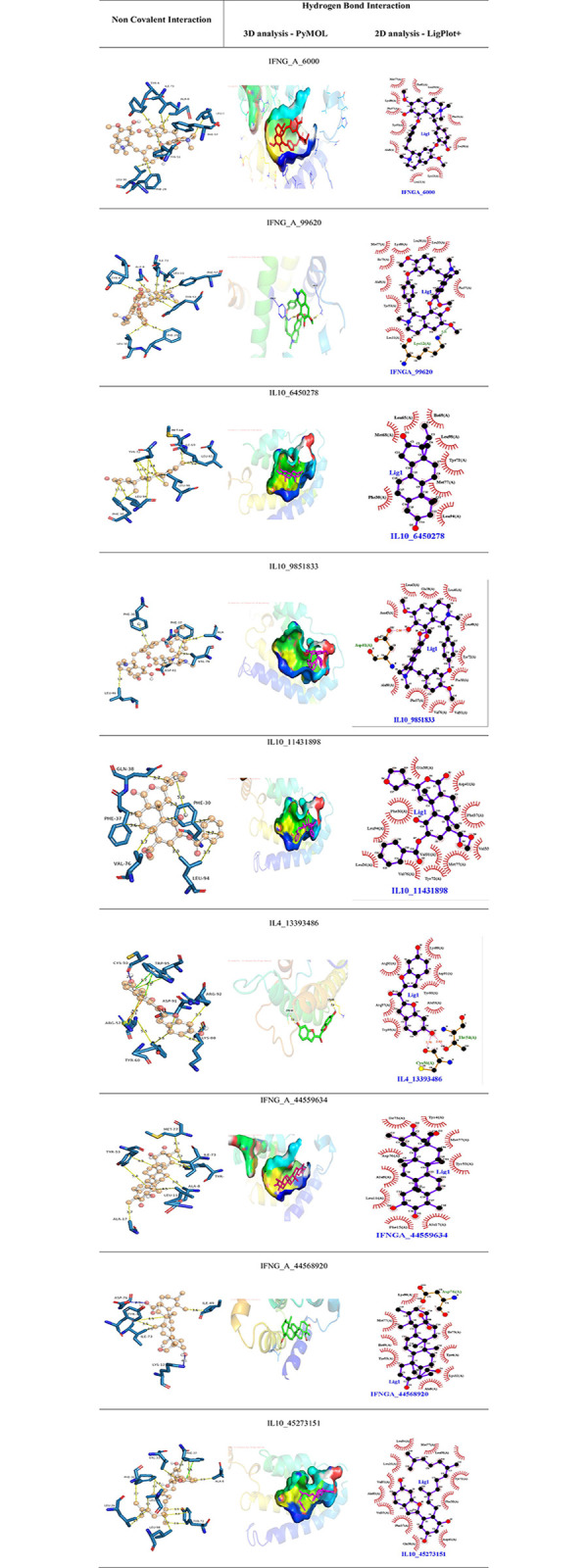
Protein-ligand interaction analysis. *First column shows the nonbonded interactions of the protein-ligand complexes where the ligand is represented as a ball and stick model and the protein predefined in the PLIP software; the distance between the interacted amino acid residues of the protein and the ligand is mentioned. The second column shows the hydrogen bond interactions in three-dimension using PyMOL where HBonds were measured, and without HBonds, the binding pocket was shown on the surface. The third column shows the hydrogen bond interactions in two-dimension using LigPlot+.

The top 10 hub genes were taken for docking analysis further. The 3-dimensional crystal structures of the hub genes were downloaded using the PDB database. The hub genes and their respective PDB IDs are as follows; TNF (1TNF), IL6 (1ALU), IL10 (1ILK), IL1B (1ILB), IL4 (1BBN), IFNG (1FG9), IL17A (2VXS), CSF2 (1CSG), CXCL8 (1IKL) and CD4 (1CDH). Proteins were prepared using AutoDock software. The missing residues were filled by the process of energy minimization using Swiss-PdbViewer (SPDBV). The active site residues were predicted using the CASTp server. The ligands were prepared using Open Babel. Finally, docking was done with 303 compounds in AutoDock Vina. The binding affinity scores having more negative values were considered a good score, and the protein-ligand complex has a better binding force. Finally, 9 compounds having a better binding affinity score (above -8.7) were taken for interaction analysis. Also, the complexes with zero RMSD value (Distance from best mode) were only taken (Table 4). Non-covalent interaction and Hydrogen bonding were analyzed in PLIP software (https://plip-tool.biotec.tu-dresden.de/plip-web/plip/index) and PyMOL software respectively. Non-bonded interactions of Protein-ligand complex were highlighted in Fig 7. 3D and 2D Hydrogen bond interactions were visualized in PyMOL and LigPlot**+,** respectively.

**Table 4:** Binding affinity scores for top protein-ligand complexes.

Target Name	Active site residue	Compound ID	Compound Name	Affinity (kcal/mol)
**IFNG_A** **(**1FG9**)**	GLN1, ASP2, PRO3, TYR4, LYS6, GLU7, GLU9, ASN10, LYS12, LYS13, ASN16, GLY18, THR27, LEU30, GLY31, LEU33, LYS34, TRP36, LYS37, GLU39, ARG42, LYS43, GLN46, LYS68, GLU71, THR72, GLU75, ASP76, VAL79, LYS80, PHE81, ASN83, SER84, LYS88	6000	Tubocurarine	-10.2
		99620	Homoaromoline	-10.2
		44559634	Triptocalline A	-9.4
		44568920	24-hydroxyursolic acid	-8.7
**IL4** **(1BBN)**	SER20, LEU21, GLU23, GLN24, ARG79, GLN82, LEU83, PHE86	13393486	Bis(6-hydroxybenzo[b]furan-2-yl)methanone	-8.7
**IL10** **(1ILK)**	LEU23, LEU26, ARG27, PHE30, VAL33, LYS34, PHE37, GLN38, LYS40, ASP41, GLN42, LEU43, ASN45, LEU46, LEU47, LEU48, LYS49, GLU50, LEU52, LEU53, PHE56, LEU65, MET68, ILE69, PHE71, TYR72, GLU75, VAL76, MET77, GLN79, ALA80, GLN83, VAL91, LEU94, LEU98, LEU101, LEU105	6450278	Guggulsterone	-8.8
		9851833	(R, S)-homoaromaline hydrochloride	-8.9
		11431898	Herkinorin	-9.1
		45273151	mulberrofuran W	-8.8

*The active site residues possess the catalytic function across evaluation, and the affinity score refers to the score value of all the non-bonded interactions.

*a*. *Hydrophobic interactions*. Hydrophobic interactions are the strongest among the non-covalent exchanges, and they exhibit non-polar nature unlike hydrophilic. The top 9 compounds formed hydrophobic interactions with the target showing higher affinity in this study. In this part, **Phenylalanine** (PHE) residue comes under hydrophobic classification, and at times, aromatic side-chain might help for stacking interactions; six out of nine compounds formed hydrophobic interaction with PHE: Tubocurarine and Homoaromoline in IFNG_A, Guggulsterone, (R, S)-homoaromaline hydrochloride, Herkinorin and mulberrofuran W in IL10. **Tyrosine** residue, which implies polar characteristics, is made from phenylalanine; seven out of nine compounds formed hydrophobic interaction with TYR: Tubocurarine, Homoaromoline, Triptocalline A and 24-hydroxyursolic acid in IFNG_A, Bis(6-hydroxybenzo[b]furan-2-yl) methanone in IL4, Guggulsterone and mulberrofuran W in IL10. **Alanine** has a non-polar characteristic which comes under minor hydrophobic classification and can get buried to form many types of interactions; five out of nine compounds formed hydrophobic interaction with ALA residue: Tubocurarine, Homoaromoline and Triptocalline A in IFNG_A, (R, S)-homoaromaline hydrochloride and mulberrofuran W in IL10. **Isoleucine** contains two non-H atoms linked with c-beta carbon leading to the form of rigid protein structure, and they also come under hydrophobic classification; four out of nine compounds formed hydrophobic interaction with ILE residue: Tubocurarine, Triptocalline A and 24-hydroxyursolic acid in IFNG_A, Guggulsterone in IL10. Both **Leucine** and **Valine** are very hydrophobic and have six compounds (Tubocurarine and Triptocalline A in IFNG_A and Guggulsterone, (R, S)-homoaromaline hydrochloride, Herkinorin and mulberrofuran W in IL10), and three compounds ((R, S)-homoaromaline hydrochloride, Herkinorin and mulberrofuran W in IL10) interacted with individual residues in the respective target. **Lysine** (Lys) is partially hydrophobic, and it interacts with two compounds out of nine: Homoaromoline in IFNG_A and Bis(6-hydroxybenzo[b]furan-2-yl) methanone in IL4. **Methionine** (MET) is very hydrophobic and interacts with two compounds: Triptocalline A in IFNG_A and Guggulsterone in IL10. **Aspartic acid** at most creates salt bridges or H bonds and sometimes hydrophobic interactions; two compounds formed hydrophobic interaction with ASP: Bis(6-hydroxybenzo[b]furan-2-yl) methanone in IL4 and (R, S)-homoaromaline hydrochloride in IL10. Both **Glutamine** and **Arginine** (partially hydrophobic) are polar, and each amino acid forms hydrophobic interaction with Herkinorin in IL10 and Bis(6-hydroxybenzo[b]furan-2-yl) methanone in IL4 respectively (http://www.russelllab.org/aas/).

*b*. *Pi stacking interactions*. **Tryptophan** and **Phenylalanine** contain aromatic side chains ready to have pi-stacking interaction with other small molecules. Tyrosine is also an aromatic amino acid that interacts with non-protein molecules through stacking interactions. Bis(6-hydroxybenzo[b]furan-2-yl) methanone had pi-stacking interaction with TRP residue in IL4; three compounds formed pi-stacking interaction with PHE: (R, S)-homoaromaline hydrochloride, Herkinorin and mulberrofuran W in IL10; Tubocurarine interacted with TYR residue of IFNG_A via pi-stacking interaction (http://www.russelllab.org/aas/).

*c*. *Hydrogen bond interactions*. Hydrogen bonding is essential in facilitating the molecular interaction between molecules, potentiating diverse cellular functions [39]. Hydrogen bonds form between two molecules by HBond Donor and HBond Acceptor, thus stabilizing the interaction between the complexes formed. In the case of **Lysine**, Homoaromoline and 24-hydroxyursolic acid created HBond with LYS residue of IFNG_A and Bis(6-hydroxybenzo[b]furan-2-yl) methanone formed HBond with LYS residue of IL4; In case of **Aspartic acid,** 24-hydroxyursolic acid formed HBond with ASP residue of IFNG_A; In case of **Cysteine**, Bis(6-hydroxybenzo[b]furan-2-yl) methanone formed HBond with CYS residue of IFNG_A [40].

*d*. *Pi-cation interactions*. This is one of the most robust interactions among the non-covalent interactions. **Arginine** is aromatic and can form pi-cation interaction with small molecules. The compound Bis(6-hydroxybenzo[b]furan-2-yl)methanone created pi-cation interaction with ARG residue of IL4.

### 3.6. Interaction analysis for compound-target network

**Fig 8:**
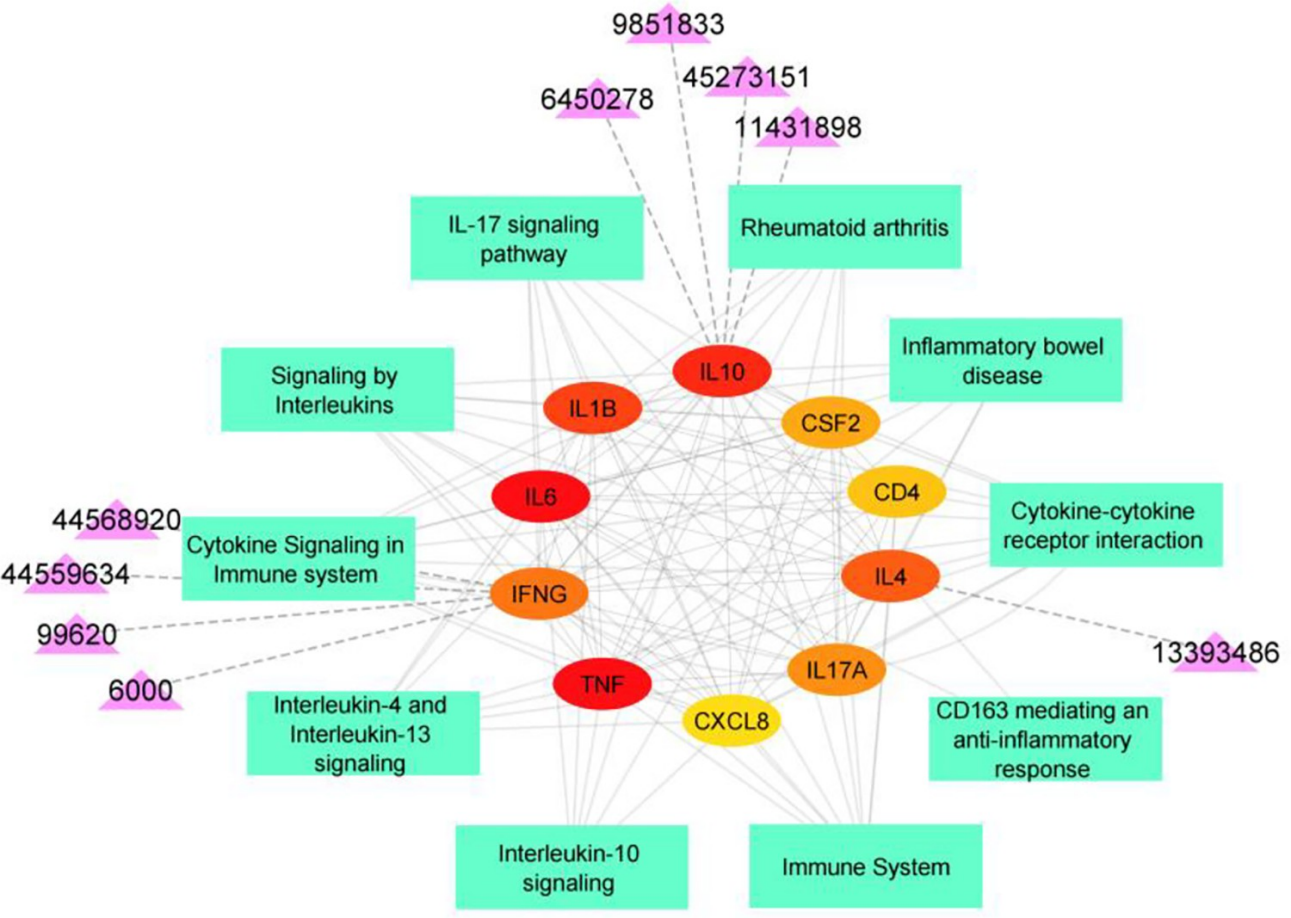
Compound-target-pathway network constructed in cytoscape. *Ellipse-shaped nodes represent ten hub genes. Rectangle-shaped nodes represent the pathways involved by the genes. Triangle-shaped nodes represent the top nine compounds, and the dotted line represents the interaction between the nine compounds with the genes IFNG, IL10 and IL4.

Network construction was done for top hub genes with their pathways and top interacting compounds. Fig 8 shows the interaction between them based on the data obtained from the above results.

Also, the topological analysis for the given network was done using Network Analyzer. It computes the topological parameters of the network. Two different results were retrieved as Summary statistics and Node Specific Statistics. The Summary Statistics table has been given in S6 File and Node Specific Statistics has been given in Table 5. IL10 gene has a high degree value of 21 with betweenness and closeness centrality.

**Table 5:** Node specific statistics of network analyzer, given topological parameters for each hub gene.

Node Specific Statistics						
**Name**	**Cluster**	**Node Status**	**Degree**	**Betweenness Centrality**	**Closeness Centrality**	**Topological Coefficient**
**IFNG**	Cluster 2	Clustered	21	0.283264046	0.777777778	0.454166667
**IL10**	Cluster 2	Clustered	21	0.312049635	0.8	0.430555556
**IL6**	Cluster 2	Clustered	19	0.058717561	0.756756757	0.439849624
**IL1B**	Cluster 2	Clustered	18	0.026971529	0.736842105	0.462301587
**TNF**	Cluster 2	Clustered	18	0.026971529	0.736842105	0.462301587
**CXCL8**	Cluster 2	Clustered	17	0.022253716	0.717948718	0.476890756
**IL17A**	Cluster 1	Clustered	17	0.01899093	0.717948718	0.478991597
**IL4**	Cluster 2	Clustered	17	0.084448224	0.717948718	0.481481481
**CSF2**	Cluster 2	Seed	16	0.01659738	0.7	0.493303571
**CD4**	Cluster 2	Clustered	13	0.001587302	0.651162791	0.557692308

*From the above-given table, IL10 has been highlighted. All of the nodes, except for IL17A, are from cluster 2, and CSF2 is the seed node.

### 3.7. MD Simulation

For the given input PDB file the command ‘pdb2gmx’ select the specific force field (charmm36) and fills the missing atoms. It generated three different format files automatically: (1).gro file–It consists of defined force fields; (2).top file–It consists of non-bonded and bonded parameters; (3).itp file–It contains information of restrain position of heavy atoms. The generated ‘.top’ file will be updated in further simulation steps. After protein topology, four ligands topology files were created using CGenFF server: (1).itp file–Ligand topology file; (2).prm file–Ligand dihedral parameters file; (3) ini.pdb file–Ligand structure file; (4).top file. For both protein and ligand topologies,.gro files will be generated as output and these files were combined for protein-ligand complex topology generation. In cubic box generation, Protein will be centered in cubic box, that is, after running the respective command the protein has been embedded in the box. The water molecules (solvent system) were added. To neutralize the system the model was checked whether it was positively or negatively charged. SOD atoms and CLA atoms were selected based upon the type of charge the system has been distributed in protein. The command ‘neutral’ will automatically recognize and neutralize the molecule by replacing its H_2_O molecule. After completely neutralizing the system, the input file ‘minim.mdp’ was given and energy minimization was done using ‘em’ command. Initially equilibration was done to the whole system before going to actual simulation step; else it will disintegrate the system and may give false results. The input file ‘nvt.mdp’ was given and temperature was stabilized (for 100ps) using ‘nvt’ command. The input file ‘npt.mdp’ was given to achieve complete pressure constant using ‘npt’ command. Finally, MD simulation was done by changing runtime (nsteps = 100ns) in ‘md.mdp’ file.

Calculating the RMSD of backbone atoms is the widely used method for checking the stability of the protein with respect to the initial structure. Generally RMSD values used to compare the docked conformation with the referenced conformation where RMSD value between 1-3Å is considered.

**Fig 9:**
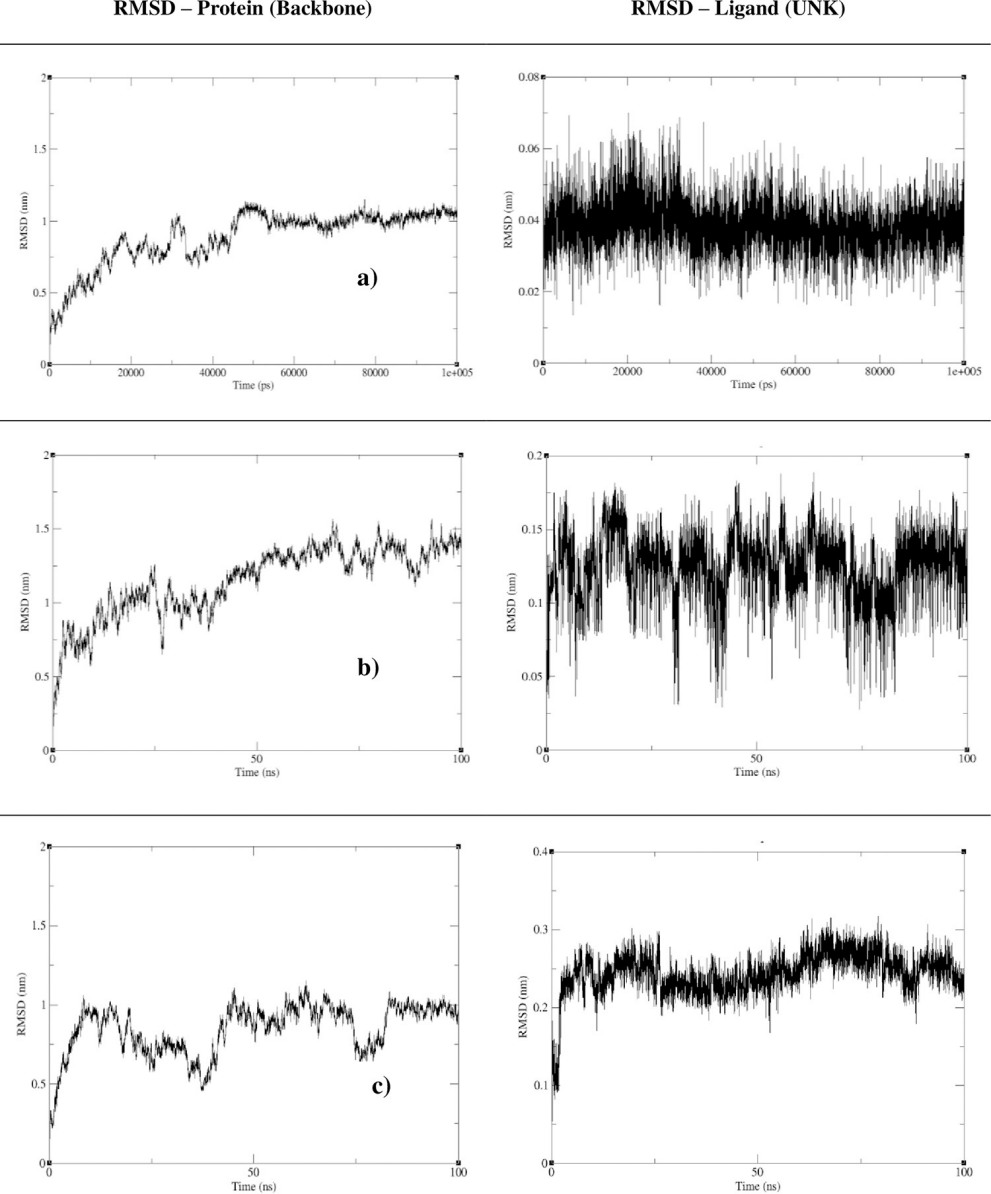
The RMSD plot of IL10 backbone and the ligand complexes. a) RMSD results of IL10 and Guggulsterone; b) RMSD results of IL10 and Herkinorin; c) RMSD results of IL10 and mulberrofuran W.

**Fig 10:**
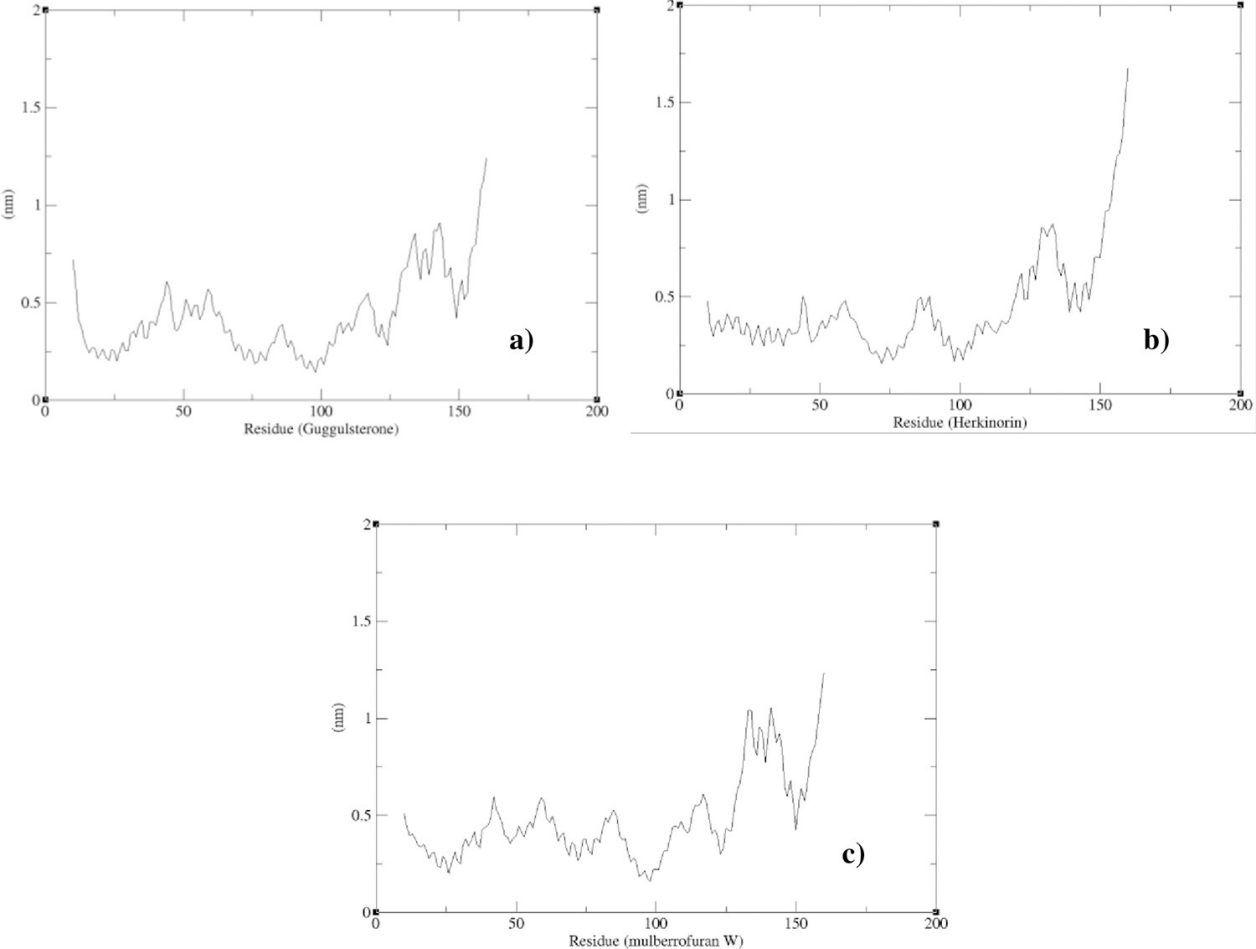
The RMSF plot results. a) IL10 and Guggulsterone; b) IL10 and Herkinorin; c) IL10 and mulberrofuran W.

In this study, the interactions of ligands with IL10 were taken for MD simulation. Out of four ligands interacted with IL10, for (R,S)-homoaromaline hydrochloride compound had no scientific evidences regarding its functions, hence it is not considered for MD simulation. Fig 9 explains the RMSD results for the remaining three compounds (Guggulsterone, Herkinorin and mulberrofuran W) and the RMSD of backbone atoms of IL10. From the results obtained, the RMSD value reached 10Å for backbone conformation; the RMSD value is less than 1Å for ligand conformation. The RMSD value has very large deviation in the beginning in all three backbone conformation. In interaction with Guggulsterone, after certain period of time (from 50 ns) the backbone RMSD reaches the equilibrate state and fluctuations. In interaction with Herkinorin and mulberrofuran W, backbone RMSD had not reached equilibration state and fluctuation and was in constant dynamic state. Also the RMSD results of ligands shows that, Guggulsterone is considerably stable with respect to IL10 structure when compared to Herkinorin and mulberrofuran W stability. Despite of the above results, the RMSD of the backbone atoms for all the three results showed major deviation in the protein. Hence the protein showed major conformational change in the structure and the ligands might not be stable with the protein binding pocket. The local fluctuations in the protein RMSF results were shown in the Fig 10. The most fluctuated regions of the protein during the simulation were indicated in the resulted plots for each of the ligand molecules. The fluctuations were observed in both the ends of the protein than in the middle regions.

From the RMSF results, the interactions with all the three ligand molecules showed more fluctuations in the protein. These results showed that the protein secondary structure elements might be less rigid in complex state with the respective ligand molecules.

## 4. Discussion

In our study, most of our targets formed hydrophobic interactions with the compounds, followed by Pi-stacking interaction, Pi-cation interaction and Hydrogen bond interaction. Most of the compounds have a hydrophobic core or non-polar. This is the reason for the interaction with the non-polar residues of the target proteins. The same condition is applicable in the case of partial polar residues in targets with compounds. Top protein-ligand interactions, IFNG-A with Tubocurarine (-10.2), Homoaromoline (-10.2), Triptocalline A (-9.4), 24-hydroxyursolic acid (-8.7); IL4 with bis(6-hydroxybenzo[b]furan-2-yl)methanone (-8.7); IL10 with Guggulsterone (-8.8), R,S-homoaromaline hydrochloride (-8.9), Herkinorin (-9.1), Mulberrofuran W (-8.8).

**IFNG-A** plays a significant role in anti-viral, anti-microbial and anti-tumor activity in humans. Typically, T-cells or Nature Killer cells produce the type II interferon for activating the immune system, thus enhancing the antigen [41, 42]. PubChem evidence suggests that *Tubocurarine*, despite having a top, binding score with the target, has toxic nature and hence it is neglected in our result. *Homoaromoline* compound is an alkaloid and presented in the roots of *Thalictrum lucidum L*. It has anti-microbial activity against *Mycobacterium smegmatis* [43]. *24-hydroxyursolic acid* has chemo-preventive nature and is presented in the *Diospyros kaki* (Persimmon) plant. It stimulates AMP-activated protein kinase (AMPK), thus inhibiting the cyclooxygenase activity (COX-2) [44].

**IL4** plays a part of the role in B-cell activation. Also, it stimulates the expression of MHC II molecules on quiescent B-cells, thus increasing the expression of immunoglobulin molecules such as IgE and IgG1 [45]. *Bis(6-hydroxybenzo[b]furan-2-yl)methanone* is a synthetic compound, but no complete information is available on the compound [46].

**IL10** plays a role in an anti-inflammatory function where it binds with the heterotetrameric receptor. The heterotetrameric receptors are IL10RA and IL10RB, which activates the JAK1 and STAT2 [47]. It restricts the antigen-presenting cells, thus reducing the expression of MHC-II and co-stimulatory molecules owing to inhibiting the T cell activation [48]. *Guggulsterone* compound properties suggest anti-inflammatory function, anti-hyperlipidemic, anti-oxidant, immunomodulatory and craving stimulus activity. It is a bioactive compound present in plants such as *Commiphora mukul* and *Commiphora wighti*. Our study suggests that in the oral, TPSA, Blood-Brain Barrier (BBB) penetration and absorption properties are interconnected. Clinical studies supported that Guggulsterone reduces the cholesterol level in the body. It has a tremendous potential immunomodulatory effect, yet *in vitro* studies are insufficient to validate the results regarding Guggulsterone. But our *in-silico* study strongly supports that Guggulsterone is the potential compound for the IL10 gene [49]. Literature evidence is not available for *(R*, *S)-homoaromaline hydrochloride*. Up-to-date, no research works were documented on *(R*, *S)-homoaromaline hydrochloride* compound. *Herkinorin* is the first semisynthetic analog of Salvinorin A. Salvinorin A present in the *Salvia divinorum* (Lamiaceae) plant. *Herkinorin* has anti-nociceptive properties and has been evaluated in a study elucidating inflammatory aches in rats [50]. *Mulberrofuran W* is a bioactive compound present in the Morus plant. It has anti-hyperglycemic activity via inhibiting the tyrosine phosphatase and anti-carcinogenic activity via inhibiting the hypoxia-activated HIF-1 α [51].

Target gene and miRNA network study implies that IL10 attained 1^st^ rank on betweenness and degree score. We suggest that the target IL10 interacted well with most of the compounds, especially with Guggulsterone having a good binding affinity score (-8.8). Guggulsterone has anti-inflammatory and immunomodulatory properties for enhancing the host immune system against COVID-19 infection. In Target-miRNA network study, IL10 associated with hsa-let-7c-5p, hsa-mir-19a-3p, hsa-mir-21-5p, hsa-mir-27a-3p, hsa-mir-98-5p, hsa-mir-106a-5p, hsa-mir-107, hsa-mir-34a-5p, hsa-mir-194-5p, hsa-mir-155-5p, hsa-mir-503-3p, hsa-mir-671-5p, hsa-mir-210-3p, hsa-mir-30d-5p. These associated miRNAs have been linked with the following diseases such as brain-related diseases, cardiovascular diseases, cancer (gastric, esophageal, lung, cervical, pancreatic, bladder, breast, colon, endometrial, prostate, skin, thyroid and other types of cancer), diabetes, epilepsy, kidney disease and several types of viral infections.

From the functional annotation results, other diseases related to the genes have been directly or indirectly linked with the COVID-19 infection. IL10 have found to be associated with conditions such as Leishmaniasis (Visceral, Cutaneous, and Urban cutaneous), Colitis, Arthritis (Experimental, Adjuvant-induced and Collagen-induced), Inflammation, Bright Disease, Glomerulonephritis, Allergic reaction, Hypersensitivity and Rheumatoid Arthritis have been highlighted in this study. IFN-gamma production is the protective immune response against the COVID-19 infection and the resultant Natural Killer cells and CD8+ T-cell activation. At the same time, IFNG is the Th1 immune response against Leimaniasis; a case study also reported co-infection of Leimaniasis with COVID-19 in an immunocompromised patient [52]. A case study regarding the GI system proposed that Ulcerative colitis has been diagnosed in a patient with diarrhea and abdominal pain following post-treatment of COVID-19 pneumonia. Data suggests that COVID-19 might influence the GI system by ACE2 protein expression [53]). One of the most common symptoms occurring in COVID-19 patients is arthralgia. However, COVID-19 might attack the musculoskeletal system of the host in its infection or post-infection stage, leading to inflammatory arthritis, but rheumatoid manifestations are yet to be confirmed [54]. Undeniably, smokers seem to be more susceptible to developing cancer and lung diseases and are more vulnerable to COVID-19 infection and COVID-19 pneumonia because smoking can increase ACE2 protein expression, leading to the entrance of the SARS-CoV-2 virus into the host more quickly than usual [55]. The conformational changes were more in the protein backbone after protein-ligand complex interaction. Though the results of MD simulation for IL10 protein and ligand molecule complexes might not showed favorable results, the functions of the protein cannot be interpreted only based on the computational analyses.

## 5. Conclusion

Overall, our study identified many of the similar compounds showing interactions between target genes; but the Guggulsterone has reached top position among all the interactions and comparing with the previous literatures. However the MD simulation results were not promising regarding protein-ligand complexes, we cannot rely only on the computational results and might do experimental studies in future to validate the functions of IL10 and Guggulsterone in COVID severe cases. Based on this whole analysis, we identified IL10 plays a main role in anti-inflammatory function and which reacts to Guggulsterone in SARS-CoV-2. The same IL10 attained the top position in miRNA network study and integrated network analysis. In specific, we recommend the compound Guggulsterone for the target IL10 against COVID-19 infection. Utterly, this is the first in silico study conducted with Guggulsterone against IL10 in COVID-19 infection. So, we hope that in future *in vitro* studies might confirm our study.

## Supporting information

S1 File (DOCX)

S2 File (DOCX)

S3 File (DOCX)

S4 File (DOCX)

S5 File (DOCX)

S6 File (DOCX)

## Abbreviations

KSK: Kabasura Kudineer
ARDS: Acute respiratory distress syndrome
IMPPAT: Indian Medicinal Plants, Phytochemistry and Therapeutics
TTD: Therapeutics Target Database
PPI: Protein-Protein Interaction
MCODE: Molecular Complex Detection
DAVID: Database for Annotation, Visualization and Integrated Discovery
SPDBV: Swiss-Pdb Viewer
MCC: Maximum Clique Centrality
GO: Gene Ontology
BP: Biological Process
CC: Cellular Component
MF: Molecular Function
GROMACS: Groningen MAchine for Chemical Simulation)
CGenFF: CHARMM General Force Field
RMSD: Root Mean Square Deviation
RMSF: Root Mean Square Fluctuation
SOD: Sodium ion
CLA: Chlorine ion
IL10: Interleukin-10
BBB: Blood-Brain Barrier
IFNG-A: Interferon gamma-A chain
IL4: Interleukin-4
ACE2: Angiotensin-converting enzyme 2
