# Supplementary material for: Interleukin-10 as Covid-19 biomarker targeting KSK and its analogues: Integrated network pharmacology
Source: PLoS One. 2023 Mar 29;18(3):e0282263. doi: 10.1371/journal.pone.0282263 (PMC10057793; doi:10.1371/journal.pone.0282263)
Supplement: S1 File — (DOCX) [file pone.0282263.s001.docx]

**Suppl. Table 1a:** Total number of active components in KSK plants. This table highlights the active components present in 15 medicinal plants of KSK.

| **S.No** | **KSK**  **Plant Name** | **Active Compounds Present** |
| --- | --- | --- |
| 1 | *Zingiber officinale* | [(2R,4R,5S,6R)-3,3,4,5-tetrahydroxy-2-propoxy-6 [[(2S,3S,4S,5R,6R)-3,4,5-trihydroxy-6-(hydroxymethyl) oxan-2-yl]oxymethyl]oxan-4-yl] (Z)-octadec-9-enoate, Angelicoidenol, cis-Sesquisabinene hydrate, (-)-camphene, (-)-Linalool, (?)-10-Epizonarene, (+)-alpha-phellandrene, (+)-Sabinene, (E)-.beta.-Farnesene, (S)-3alpha-[(S)-1,5-Dimethyl-4-hexenyl]-6-methylenecyclohexene, [4]-Gingerdiacetate, [6]-Gingerdiol 3,5-diacetate, 1-(4-Hydroxy-3-methoxyphenyl)-3,5-octanediol, 1-(4-hydroxy-3-methoxyphenyl)tetradecane-3,5-diol, 1-Dehydro-6-gingerdione, 1-Nonanol, 3-Methylbutanal, 6-Gingerdiol, 6-Gingesulfonic acid, 6-Shogaol, alpha-Farnesene, alpha-TERPINEOL, beta-Bisabolene, BETA-PHELLANDRENE, BETA-PINENE, beta-Sesquiphellandrene, Citral, Dehydro-10-gingerdione, Dehydrozingerone, Dipentene, DITERPENE II (LACTONE), DL-Arginine, DL-ASPARTIC ACID, DL-Valine, ent-Zingiberene, Eucalyptol, GAMMA-TERPINENE, GERANIOL, GERANYL ACETATE, Gingerdiol, Gingerdione, Gingerenone A, Gingerenone B, Gingerenone C, GingerglycolipidA,Gingerglycolipid B, gingerol, glycine, HEPTANE, Hexahydrocurcumin, Isogingerenone B, l-alpha-Curcumene,L-cysteine, l-isoleucine, L-leucine, L-serine, L-threonine,L(-)-Borneol, Methyl-[12]-Gingediol, MYRCENE, nerol, octane, sesquithujene, Vanillylacetone. |
| 2 | *Piper longum* | isobutyl amide, Phytosterols, (2E,4E)-N-Isobutyl-2,4-decadienamide, 2-PHENYLETHANOL, 4'-Methoxyacetophenone, 6,7-dibromo-4-hydroxy-1H,2H,3H,4H-pyrrolo[1,2-a]pyrazin-1-one, alpha thujene, Aristololactam, BETA-CARYOPHYLLENE, Diaeudesmin, Dihydrocarveol, EICOSANE, Fargesin, guineensine, HENEICOSANE, HEPTADECANE, HEXADECANE, l-asarinin, Lignansmachilin F, Methyl 3,4,5-trimethoxycinnamate, NONADECANE, OCTADECANE, P-CYMENE, piperine, Piperlongumine, Piperlonguminine, PIPERNONALINE, Piperundecalidine, PLUVIATILOL, TERPINOLENE, TRIACONTANE. |
| 3 | *Syzygium aromaticum* | (4Z)-4,11,11-trimethyl-8-methylidenebicyclo[7.2.0]undec-4-en-7-ol, 1-Methylhexyl acetate, 1,5-Anhydro-1-(5,7-dihydroxy-2-methyl-4-oxo-4H-chromen-8-yl)hexitol, 10,11-dihydroxy-2,2,6a,6b,9,9,12a-heptamethyl-1,3,4,5,6,6a,7,8,8a,10,11,12,13,14b-tetradecahydropicene-4a-carboxylic acid, 2-HEPTANOL, 2-HEPTANONE, 2-hydroxy-4,6-dimethoxy-5-methylacetophenone, 2-NONANOL, 2-NONANONE, 2-Nonyl acetate, 2,3-Digalloylglucose, Acetyleugenol, Biflorin, Caryophyllone Oxide, Casuarictin, Eugeniin, Eugenin, eugenol, heptan-2-one, heptan-2-yl benzoate, Isoeugenitol, naphthalene, Pentagalloyl Glucose, SCHEMBL6761380, Syzyginin A, Syzyginin B |
| 4 | *Hygrophila auriculata* | 3beta-Hydroxy-20(29)-lupene, Isoflavone glycoside, Xylan,Birch, 3-Methylnonacosane, Apigenin 7-glucuronide, BETA-D-XYLOPYRANOSE, Betulin, ethanol, l-ascorbic acid, L-histidine, Lipase, nicotinate, octadeca-9,12-dienoic acid, Octadecanoate, oleic acid, palmitic acid, phenylalanine, Potassium, S-2,6-diaminohexanoic acid, sterol. |
| 5 | *Terminalia chebula* | AC1L4F91, Tea extract, 2-Coumarinate, 2,4-Dihydroxycinnamic acid, acyclovir, Arachidic acid, arjungenin, arjunglucoside I, Arjunolic acid, Bellericoside, BIS (ACETIC ACID); TANNINS, Chebulagic acid, Chebulinic acid, Daucosterol, DL-Asparagine, Docosanoic acid, ETHYL GALLATE, ferulic acid, Gallic acid, Hyptatic acid A, L-Arabinose, phloroglucinol, Punicalagin, pyrogallol, shikimic acid, TANNIC ACID, Terchebin, Terchebulin, Terminolic acid, TRIACONTANOIC ACID, UNII-I76294A13O, Vanillic acid. |
| 6 | *Saussurea costus* | 11,13-Epoxydehydrocostuslactone, alpha-Cyclocostunolide, Inulin from chicory, .alpha.-Elemene, (+)-germacrene A, 1,3-dibutyl-4-methylbenzene, 1,8,11,14-Heptadecatetraene, Alantolactone, alpha-Costol, ALPHA-IONONE, ALPHA-SELINENE, beta-Cyclocostunolide, beta-Ionene, BETA-SELINENE, Costic acid, Costol, Costunolide, Dehydrocostus lactone, DIOSGENIN, Friedlein, gamma-Costol, germacra-1(10),4,11(13)-trien-12-ol, I-22,23-Dihydrostigmasterol, Isoalantolactone, Isodehydrocostus lactone, Isozaluzanin C, Picrotoxinum, Saussurea lactone, Saussureamine A, Saussureamine B, Saussureamine C, Taraxasterol, trans-alpha-Bergamotene. |
| 7 | *Tinospora cordifolia* | 1-Heptacosanol, 1-Octacosanol, 15-Nonacosanone, berberine, cardiofolioside B, Chasmanthin, Columbin, Cordifolide A, Magnoflorine, Palmarin, Tembetarine, Tinosponone, Tinosporinone, xanosporic acid. |
| 8 | *Clerodendrumserratum* | triterpenoids, AC1LAVB6, apigenin, baicalein, eupatin, luteolin, OLEANOLIC ACID, Queretaroic acid, Scutellarein, Serratagenic acid, Verbenalin. |
| 9 | *Andrographis paniculata* | 14-Deoxyandrographoside, Dehydroandrographoline, MLS001143515, Stigmasta-5,22-dien-3-ol, 14-Deoxy-11-oxoandrographolide, 14-Deoxy-11,12-didehydroandrographolide, 14-deoxyandrographolide, 3-O-caffeoyl-D-quinic acid, 5-Hydroxy-3,7,8-trimethoxy-2-(2-methoxyphenyl)-4H-chromen-4-one, 5-Hydroxy-7,8,2'-trimethoxyflavone 5-glucoside, 5-hydroxy-7,8,2',3'-tetramethoxyflavone, andrograpanin, Andrographidine A, andrographidine B, Andrographidine C, andrographidine D, Andrographidine E, andrographidine F, Andrographin, Andrographiside, andropanoside, CARVACROL, CHEMBL479285, Citrostadienol, Deoxyandrographolide. |
| 10 | *Cyperus rotundus* | beta-Rotunol, Mustakone, UNII-0V56HXQ8N5, (-)-Rotundone, 3-Carene, 4-METHOXYBENZOIC ACID, alfa-Cyperone, alpha-rotunol, Arsenic, Aureusidin, beta-Cyperone, Calcium, Copadiene, Copper, Cyperene, Cyperenone, Cyperol, Cyperolone, Cyperotundone, glycerol, Iron, Isocyperol, Isokobusone, Kobusone, linolenic acid, Magnesium, Nickel, quercetin, resmethrin, Scoparone, Sesquiterpene II alcohol diglucoside (Compound 1), Tetradecanoic acid. |
| 11 | *Anacyclus pyrethrum* | Anacyclin, dodeca-2E,4E-dienoic acid isobutylamide, Isobutyramide, Lidocaine hydrochloride, Pellitorine. |
| 12 | *Tragia involucrata* | vinylhexylether, shellsol, 2,4-dimethyl hexane, 2-methylnanone, 2,6-dimethyl heptane, stigmasterol, rutin. |
| 13 | *Justicia adhatoda* | Vasicine, vasicinone, adhavasinone , vasicinolone, adhavasine, adhatodine, anisotine, vasicoline |
| 14 | *Coleus amboinicus* | Cirsimaritin, oxalacetic acid, Thymol. |
| 15 | *Sida acuta* | Ecdysterone, .alpha.-Amyrin, betaine, choline, Cryptolepine, Hypaphorine, Peganine, Phenethylamine, PSEUDOEPHEDRINE, Vasicinol, |

**Suppl. Table 1b:** Total Compounds (KSK phytocompounds and KSK similar compounds) count – 1245 compounds

**Total Compounds (KSK phytocompounds and KSK similar compounds) count – 1245 compounds**

24(S)-hydroxycholesterol

27-hydroxycholesterol

22R-hydroxycholesterol

25-hydroxycholesterol

7alpha-hydroxycholesterol

7alpha,27-dihydroxycholesterol

7beta-hydroxycholesterol

7-hydroxycholesterol

7beta, 27-dihydroxycholesterol

7beta, 25-dihydroxycholesterol

7alpha,25-dihydroxycholesterol

HE2100

Lanosterol

LAWSARITOL

(24S)-ethylcholesta-7,9(11),22(E)-triene-3b-ol

24(S), 25-epoxycholesterol

HALOXYSTEROL A

Aloradine

Pregnenolone

Organon

Ethylestrenol

Dihydrotachysterol

Ergocalciferol

Olean-12-en-3beta,15alpha-diol

Secalciferol

LUPEOL

Eupatilin

CHRYSOERIOL

2,6-dihydroxy-1,7-dimethoxyxanthone

CENTAUREIDIN

SMEATHXANTHONE A

HERBACETIN

ISORHAMNETIN

TAMARIXETIN

BAICALEIN

SCUTELLAREIN

GOSSYPETIN

NSC-106970

7,8,3',4'-tetrahydroxyflavone

6,7-Dihydroxy-2-phenyl-chromen-4-one

GARTANIN

MANGOSTIN

Hesperetin

HOMOERIODICTYOL

3-O-METHYLQUERCETIN

3,4-dihydroxyxanthone

GARCINONE D

8-DEOXYGARTANIN

Nobiletin

2-(3,4-Dihydroxy-phenyl)-7-hydroxy-chromen-4-one

2-(3,4-Dihydroxy-benzyl)-7-hydroxy-chromen-4-one

MANGOSTANIN

PERSICARIN

5-deoxyabyssinin II

3-methoxy-4-hydroxylonchocarpin

RHODIOLININ

Cudraxanthone D

Cudraxanthone L

Gamma-mangostin

CYANIDIN

3,7,3',4'-TETRAHYDROXYFLAVONE

ROBINETIN

7,8,4'-trihydroxyisoflavone

2-D08

MANGOSTENONE F

MANGOSTENONE G

CUDRATRICUSXANTHONE

Isorhamnetin 3,7-disulfate

Abyssinin II

OCHNAFLAVONE

CYANIDIN CHLORIDE MONOHYDRATE

8-prenylquercetin

ERIODICTYOL

Myricetin

CIRSIMARIN

ZAPOTIN

Delphinidin

Cyrtominetin

MANGOSTANOL

Abyssinin I

SIGMOIDIN B

7,3',4'-trihydroxyisoflavone

Silymarin

Silibinin

MANGIFERIN

Gallic acid 5,6-dihydroxy-3-carboxyphenylester

2-(3-hydroxyphenyl)-7-methoxychroman-4-one

WEDELOLACTONE

2'-hydroxy-3,4,5-trimethoxychalcone

MACLURAXANTHONE

ACACETIN

Sigmoidin F

Cudraxanthone M

PHENSTATIN

BNC-105

ARTORIGIDIN A

KAEMPFERIDE

7-Hydroxy-2-(4-methoxy-benzyl)-chromen-4-one

2-(4-Hydroxy-phenyl)-7-methoxy-benzofuran-5-ol

LIM-0705

PELARGONIDIN CHLORIDE

SULFURETIN

ARTOBILOXANTHONE

7-hydroxy-2-(3-hydroxyphenyl)chroman-4-one

Kaempferol-3-O-methyl ether

BAICALIN

PHLOROFUCOFUROECKOL A

Diosmin

Isorhamnetin 3-O-rhamnoside

FUKUGETIN

SIGMOIDIN A

6-methoxykaempferol 3-O-beta-D-robinobioside

Isogemichalcone C

Hidrosmin

CIRSIMARITIN

WOGONIN

EUPAFOLIN

QUERCITRIN

3,7-Bis(2-hydroxyethyl)icaritin

CONTIGOSIDE B

ICARITIN

SOPHOFLAVESCENOL

MRS1093

Tamarixetin 3-glucoside-7-sulfate

Rutin

Quercetin 3-O-neohesperidoside

Broussoflavonol F

6-tert-butyl-m-cresol

2-tert-butylbenzene-1,4-diol

4-Methyl-1,1':4',1''-terphenyl-3,4''-diol

2,6-di-t-butylphenol

Cannabidiol

GWP42006

9-phenanthrol

6-(3-Hydroxy-phenyl)-naphthalen-1-ol

DuP-654

4-Fluoro-1,1':4',1''-terphenyl-3,3''-diol

HEXESTROL

CP-4497

4-Iodo-2,6-diisopropyl-phenol

6-(4-Hydroxy-phenyl)-1-methyl-naphthalen-2-ol

3-(2-naphthyl)phenol

Naphthalene-1,4-diol

5,3'-Dipropyl-biphenyl-2,4'-diol

4'-(trifluoromethyl)-4-biphenylol

EFFUSOL

6-(4-Hydroxy-phenyl)-naphthalen-1-ol

6-(2-Hydroxy-phenyl)-naphthalen-2-ol

1,1':4',1''-terphenyl-3,3''-diol

Medrysone

Prednisolone

Fludrocortisone

Triamcinolone

UVAOL

ME-3738

OLEANOLIC_ACID

URSOLIC ACID

3beta-hydroxyrus-12,19(29)-dien-28-oic acid

androstenol

3beta-hydroxyurs-12-en-27-oic acid

Methyl 3beta-hydroxyolean-12-en-28-oate

3beta-hydroxyolean-12-en-27-oic acid

Cholesterol

BETULIN

Sitosterol

desmosterol

Myrrhanol A

2alpha-Hydroxyolean-12-en-28-oic acid

2alpha-Hydroxyurs-12-en-28-oic acid

2-isooleanolic acid

2-isoursolic acid

CHOLINE IODIDE

triethylcholine

CP-339818

[1-Benzyl-1H-quinolin-(4E)-ylidene]-hexyl-amine

[2-(3H-Indol-1-yl)-ethyl]-dimethyl-amine

NSC-306843

L-Tryptophan

D-tryptophan

NOX-200

6-fluorotryptophan

1-methyl-L-tryptophan

Apo805K1

indole-3-propionic acid

Golotimod

PO3 2-Nle-Trp-O-3K

IRL-1722

PO3 2-Ile-Trp-O-3K

PO3 2-Leu-Trp-O-3K

Oglufanide

lisinopril-tryptophan

L-Tryptophan-L-2-aminoadipic acid

L-Tryptophan-L-leucine

L-Tryptophan-L-arginine

L-Tryptophan-L-glutamine

L-703014

IRL-1841

L-708568

L-Tryptophan-L-aspartic acid

L-Tryptophan-L-asparagine

NSC-401077

Desoxypeganine

Amphetamine

Dextroamphetamine

R(-)amphetamine

2,2-Diphenyl-ethylamine

P-IODOAMPHETAMINE

Phenelzine

Methamphetamine

Tranylcypromine

Cis-2-phenylcyclopropylamine

3,3-diphenylpropan-1-amine

MOFEGILINE

Phentermine

Trans-2-fluoro-2-phenylcyclopropylamin

Cis-(+/-)-2-Fluoro-1,2-diphenylcyclopropylamine

Cis-2-fluoro-2-phenylcyclopropanamine

(S)-2-Amino-3-phenyl-propane-1-thiol

(1-phenylcyclopentyl)methanamine

1,2,3,4-Tetrahydro-naphthalen-2-ylamine

Phenethyl-(3-phenyl-propyl)-amine

Phenethyl-(4-phenyl-butyl)-amine

LJP-1207

(S)-2-Amino-4-phenyl-butane-1-thiol

norfenfluramine

C-(5H-Dibenzo[a,d]cyclohepten-5-yl)-methylamine

(+)-norfenfluramine

Trans-2-fluoro-2-(4-fluorophenyl)cyclopropanamine

Cis-2-(para-fluorophenyl)cyclopropylamine

(-)-norfenfluramine

2-(2'-methyl-biphenyl-3-yl)-ethylamine

Mephentermine

4-phenyl-1,2,3,6-tetrahydropyridine

1-biphenyl-2-ylmethanamine

N'-(2-phenylallyl)hydrazine hydrochloride

Phenformin

PEITC

(1R,2R)-1,2-diphenylethane-1,2-diamine

3-Phenyl-pyrrolidine

(1R,2S)-1,2-diphenylethane-1,2-diamine

9-(Aminomethyl)-9,10-dihydroanthracene

Trans-2-fluoro-2-p-tolylcyclopropanamine

(S)-2-Amino-2-phenyl-ethanethiol

BXT-51072

Phenylpropanolamine

MK-1496

Oxilofrine

Phenmetrazine

Sucrose

Lactulose

Hydroxyethyl starch

Pellitorin

Dodeca-2E,4E-dienoic acid isobutylamide

NPS 1776

Lidocaine

QX-314

PC-24

Etidocaine

Tocainide

Mepivacaine

Nefiracetam

Bupivacaine

Levobupivacaine

Ropivacaine

Prilocaine

IQB-9302

LIDOFLAZINE

HALOXYSTEROL B

KAEMPFEROL

GALANGIN

MORIN

3,7-dihydroxy-flavone

APIGENIN

CHRYSIN

7,4'-Dihydroxyflavone

7-Hydroxy-2-(4-hydroxy-benzyl)-chromen-4-one

NSC-94258

DIHYDROKAEMPFEROL

ISOLICOFLAVONOL

2-Benzyl-7-hydroxy-chromen-4-one

XAP044

LIQUIRTIGENIN

NSC-26745

8-prenylapigenin

NARINGENIN

7-hydroxy-2-phenylchroman-4-one

PINOCEMBRIN

(2S)-5,7,2',4'-tetrahydroxyflavanone

Hesperidin

ASTRAGALIN

ICARIIN

Icariside II

7-(2-Hydroxyethyl)-3-O-rhamnosylicariin

Apigenin-7-O-beta-D-glucuronide methyl ester

TILIROSIDE

Apigenin-7-O-beta-D-glucuronide

naringin

Daidzin

SOPHORICOSIDE

Brutieridin

Phlorizin

GERANIIN

Dosmalfate

FUROSIN

Mallotusinic acid

Mallotinic acid

Chebulagic acid

Kushenol N

Chebulinic acid

Sanguiin H-6

Abyssinoflavanone VI

2,3-dihydropyrrolo[2,1-b]quinazolin-9(1H)-imine

borneol

FARNESYL

squalene

P-hydroxyphenethyl trans-ferulate

Octahydrocurcumin

FERULIC ACID

isoferulic acid

ACEROGENIN B

Acerogenin A

Dimethylnordihydroguarierate acid

gingerol

EUGENOL

5-PENTYL-2-PHENOXYPHENOL

5-hexyl-2-phenoxyphenol

5-octyl-2-phenoxyphenol

ENDIANDRIN A

Go-Y022

Caffeic acid phenethyl ester

(-)-pinoresinol

CA4P

DEHYDROZINGERONE

(-)-thujaplicatintrimethyl ether

NSC-381864

Terameprocol

MEDIORESINOL

Curcumin

DEMETHOXYCURCUMIN

ASC-J9

Acerogenin C

Isovanillin

Go-Y026

Ethylvanillin

Docosanol

DI-O-METHYLENDIANDRIN A

(+)-3,3'-bisdemethyltanegool

N-Omega-Hydroxy-L-Arginine

Nomega-hydroxyarginine

L-NIO

L-Homoarginine

N,N-dimethylarginine

L-ornithine

D-ornithine

N5-(1-iminobutyl)-L-ornithine

N5-(1-iminopropyl)-L-ornithine

HQK-1004

Alpha-Aminobutyric Acid

L-leucine

L-isoleucine

Selenomethionine Se-75

Penicillamine

Plaunotol

CYLINDOL A

3,4-dibenzyloxy-2'-hydroxychalcone

vanillylmandelic acid

2,3-dimethoxy-2'-hydroxychalcone

Tetradecane

Syringic Acid

2,4-dimethoxy-2'-hydroxychalcone

NSC-119913

toluene

4-(trifluoromethyl)biphenyl

[(1e)-4-Phenylbut-1-Enyl]Benzene

Cysteine Hydrochloride

L-homocysteine

L-cystine

S-sulpho-L-cysteine

Double Oxidized Cysteine

S-(Dimethylarsenic)Cysteine

L-valine

threo-3-methylglutamate

2S,4R-4-METHYLGLUTAMATE

N-Methylleucine

NV-5138

4-Amino-3-hydroxy-butyric acid

Nonoxynol-9

VERAGUENSIN

Stiripentol

DIHYDROCUBEBIN

3-Phenyl-1,2-Propandiol

Benzyl alcohol

(S)-1,7-Diphenyl-6(E)-hepten-3-ol

1,1,1,2,2,3,3-heptafluoro-8-phenyloctan-4-ol

R411

1-(4-Methoxy-phenyl)-2-phenyl-ethane-1,2-dione

4-Methoxybenzaldehyde

P-Anisic Acid

Sulfamic acid 4-(3-methoxy-benzoyl)-phenyl ester

Sulfamic acid 3-(4-methoxy-benzoyl)-phenyl ester

2-methylbut-3-yn-2-yl 4-methoxybenzoate

4,5-Bis(4-methoxyphenyl)-3H-1,2-dithiol-3-one

Sulfamic acid 4-(2-methoxy-benzoyl)-phenyl ester

2-(2-Methoxy-benzoyl)-cyclohexane-1,3-dione

1-(4-hydroxyphenyl)prop-2-en-1-one

SCH-546909

(-)-3,3'-bisdemethylpinoresinol

Alpha-methylcubebin

(8R,8'R,9'S)-5-methoxyclusin

(-)-cubebinin

(-)-dihydroclusin

isopulegol

(-)-clusin

ETHOXYCLUSIN

L-652731

(-)-yatein

HINOKININ

PIPERINE

Dehydropipernonaline

PIPERNONALINE

MAACKIAIN

(8R,8'R)-4-hydroxycubebinone

Methyl piperate

PIPERROLEIN B

T-686

ASIATIC ACID

18alpha-Glycyrrhetic acid

11-keto-ursolic acid

Methyl3beta-hydroxyolean-12-en-27-oate

Pomolic acid

Spathodic acid

Rotungenic acid

3beta-hydroxyrus-18,20(30)-dien-28-oic acid

N-Butyl 2beta-hydroxyolean-12-en-28-oate

Ethyl 2beta-hydroxyolean-12-en-28-oate

3-acetyl-11-keto-ursolic acid

19alpha,24-dihydroxyurs-12-en-3-on-28-oic acid

Carbenoxolone

Oleanonic acid

Heptane-2,3-dione

octanol

1-DODECANOL

1,1,1-trifluorododecan-2-one

1,1,1-Trifluoro-undecan-2-one

1,1,1-Trifluoro-tridecan-2-one

1'-acetoxychavicol acetate

4-hexylphenyl propiolate

Abyssinoflavanone VII

Puerarin

ABYSSINONE V

8-(3-methylbutyl)naringenin

SOPHORAFLAVANONE B

(2S)-abyssinone II

8-n-propylnaringenin

8-n-pentylnaringenin

8-n-heptylnaringenin

8-n-nonylnaringenin

Sophoraflavanone G

Abyssinone-IV

KURARINONE

BURTTINONE

8-n-undecylnaringenin

8-(2-methylpropyl)naringenin

KUSHENOL A

LEACHIANONE A

(2S)-euchrenone a7

NSC-180246

8-methylnaringenin

(2S)-2'-methoxy kurarinone

8-(2,2-dimethylpropyl)naringenin

3-(4-Hydroxyphenyl)-7-isopropoxychromen-4-one

Ablukast

KURARINOL

8-benzylnaringenin

sakuranetin

ISOSAKUTANETIN

NSC-407228

FPL-55712

2-(4-hydroxyphenyl)-7-methoxychroman-4-one

(+)-Myristinin A

7-Cyclopentyloxy-3-(4-hydroxyphenyl)chromen-4-one

4-hydroxylonchocarpin

4',5,7-trihydroxy-6,8-dimethylisoflavone

Biomed 101

YM-26734

3,9-dihydroxy-2,10-diprenylpterocap-6a-ene

LY-2300559

Eryvarin D

AMENTOFLAVONE

ISODISPAR B

Kuwanon L

ISOFORMONENTIN

biochanin A

FORMONONETIN

prunetin

L-165041

DIHYDROXANTHOHUMOL

(+)-Myristinin D

3-methoxytyramine

6-(4-Hydroxy-phenyl)-1-methoxy-naphthalen-2-ol

2-methoxy-4-(2-nitrovinyl)phenol

BENZYL BENZOATE

3-(ethoxycarbonyl)phenylboronic acid

NY-008

dibutyl phthalate

Dihexan-3-yl 5-(hydroxymethyl)isophthalate

Diheptan-3-yl 5-(hydroxymethyl)isophthalate

6-Acetyl-7-hydroxy-2H-chromen-2-one

7-hydroxy-6-propionyl-2H-chromen-2-one

8-Acetyl-7-hydroxy-2H-chromen-2-one

7-hydroxy-8-propionyl-2H-chromen-2-one

KURAIDIN

Abyssinone-VI-4-O-methyl ether

OXYBENZONE

6-acetyl-7-methoxy-2H-chromen-2-one

CORDOIN

2,2',4,4'-tetrahydroxy-6'-methoxychalcone

6-acetyl-7-propoxy-2H-chromen-2-one

6-Acetyl-7-ethoxy-2H-chromen-2-one

Genistein

daidzein

AH6809

2-Benzhydryl-7-hydroxy-chromen-4-one

8-acetyl-7-methoxy-2H-chromen-2-one

7-methoxy-8-propionyl-2H-chromen-2-one

MESUAGENIN B

8-acetyl-7-ethoxy-2H-chromen-2-one

8-acetyl-7-propoxy-2H-chromen-2-one

7-ethoxy-8-propionyl-2H-chromen-2-one

LY-282210

MESUAGENIN A

3-hydroxy-4,7-dimethyl-6H-benzo[c]chromen-6-one

Trioxsalen

Beta-naphthylboronic acid

1,4-diphenyl-(1E,3E)-1,3-butadiene

1,2,3,4,6-penta-O-galloyl-beta-D-glucose

Tellimagrandin II

EUGENIIN

PUNICAFOLIN

1(beta)-O-galloylpedunculagin

tannic acid

1,2,6-tri-O-galloyl-beta-D-glucose

CORILAGIN

PEDUNCULAGIN

5-desgalloylstachyurin

CASUARIIN

GALLOCATECHIN GALLATE

N-dodecylgallate

N-cetylgallate

THEASINENSIN A

ETHYLGALLATE

4-hydroxyphenethyl 3,4,5-trihydroxybenzoate

3-hydroxyphenethyl 3,4,5-trihydroxybenzoate

Palomid-529

ELLAGIC ACID

Salvianolic acid B

BETULINIC ACID

T-1095

Rotenone

D-glucose

D-mannose

Beta-D-Glucose

Alpha-D-Mannose

Beta-D-Mannose

Alpha-D-Fucose

Beta-L-fucose

4,6-Dideoxyglucose

6-Deoxy-Alpha-D-Glucose

Fludeoxyglucose F 18

2-Deoxy-2fluoro-Glucose

Tagatose

Fructose

alpha-MDG

SGN-2FF

US8703720, Reference Compound 1

Sofalcone

23-hydroxybetulinic acid

ALPHA-FMH

carnosine

Polaprezinc

Diphthamide

Ingavirin

Prezatide copper acetate

5-methyl nicotinic acid

ISONICOTINIC ACID

Quinolinic Acid

Palmitoleic Acid

alpha-linolenic acid

Docosapentaenoic acid

Epanova

(E)-octadecan-9-ynoic acid

(Z)-7-octedecan-9-ynoic acid

(5Z,9Z)-5,9-heptacosadienoic acid

Gamolenic acid

Arachidonic Acid

RT-001

Sterculic acid

Hexadecanoic acid

stearic acid

Caprylic acid

Decanoic Acid

Lauric Acid

undecanoic acid

MYRISTIC ACID

N-Tridecanoic Acid

HEPTANOATE

IODOSTEARIC ACID

7,9-octadecadiynoic acid

Azelaic Acid

SEBACIC ACID

Heptanoic Acid

phytanic acid

pristanic acid

12-hydroxylauric acid

7,9-tetradecadiynoic acid

Rac-2-amino-4-phenylbutanoic acid

Iodo-Phenylalanine

3-Methylphenylalanine

(S)-phenylglycine

Methyl L-phenylalaninate

alpha-methylphenylalanine

L-ornithine phenylacetate

2-amino-2-(2-fluorophenyl)acetic acid

L-beta-BA

fenclonine

Beta-(2-Naphthyl)-Alanine

(S)-2-amino-2-o-tolylacetic acid

(S)-2-amino-2-p-tolylacetic acid

3-amino-5-(4-octylphenyl)pentanoic acid

2-amino-2-(2,3-difluorophenyl)acetic acid

2-amino-2-(2,4-difluorophenyl)acetic acid

4-amino-6-(4-octylphenyl)hexanoic acid

4-Amino-3-(4-fluoro-phenyl)-butyric acid

PARA-(BENZOYL)-PHENYLALANINE

LY307452

(S)-2-amino-2-phenylpropanoic acid

()-2-Aminoindane-2-carboxylic acid

L-lysine

D-lysine

Norleucine

meso-DAP

androstanol

trihydroxycholestane

3-epicorosolic acid methyl ester

Tormentic acid methyl ester

3 beta-O-acetyloleanolic acid

2-Oxoolean-12-en-28-oic acid

1,5-bis(4-hydroxyphenyl)penta-1,4-dien-3-one

Salicyclic acid

GW7604

NSC-54162

3,4-Dihydroxycinnamic Acid

2-Hydroxycinnamic acid

3-(2,4-dihydroxyphenyl)propionic acid

N-butylresorcinol

P-Coumaric Acid

OXYRESVERATROL

4-hexyl resorcinol

2,4,3',5'-tetrahydroxybibenzyl

Ganciclovir

BRL-44385

9-Methylguanine

BETA-HYDROXYETHYL THEOPHYLLINE

7-methylxanthine

MIV-210

9-octadecynoic acid

MASLINIC ACID

Corosolic acid

Augustic acid

3-epi-masilinic acid

Amooranin

3-acetyl-11-keto-beta-boswellic acid

Asiaticoside

WF-11605

GLYCYRRHIZIN

B-10

Durhamycin A

ginsenoside Rg3

dioscin

kallstroemin D

hypoglaucin A

SCH-725737

SCH-725739

L-asparagine

4,5,6-trihydroxy-3-methylphthalide

2,3,4-trihydroxybenzoic acid

ALTENUSIN

3,5-dihydroxybenzoic acid

BUTEIN

2-(3,4-Dihydroxyphenyl)Acetic Acid

1,3-bis(3,4-dihydroxyphenyl)prop-2-en-1-one

3beta,6beta-dihydroxyolean-12-en-27-oic acid

3beta-acetoxyolean-12-en-27-oic acid

RESORCINOL

PYROGALLOL

PHENOL

Hydroquinone

CATECHOL

Phloroglucinol

Shikimate-3-Phosphate

Lithospermic acid

Chromomycin a3

2-hydroxyphenethyl 3,4,5-trihydroxybenzoate

Chlorogenic acid

CALCEOLARIOSIDE A

CALCEOLARIOSIDE B

Dibenzo-p-dioxin-2-carboxylic acid

3-hydroxy-2-methoxybenzaldehyde

Isohelenin

Santonin

Lactitol

4'-GALACTOSYLLACTOSE

Seprilose

10-EPI-8-DEOXY-CUMAMBRIN B

Perillyl alcohol

DEHYDROLEUCODIN

Arglabin

CYNAROPICRIN

Picrotoxinin

1,1,1-Trifluoro-nonadecan-2-one

9-O-[2-(Phenylol-1-yloxy)ethyl]berberine bromide

9-O-[3-(Phenylol-1-yloxy)propyl]berberine bromide

9-O-[4-(Phenylol-1-yloxy)butyl]berberine bromide

9-O-[2-(Phenylol-1-yloxy)hexyl]berberine bromide

PALMATINE

9-O-[5-(Phenylol-1-yloxy)pentyl]berberine bromide

9-O-[3-(2-Pyridinoxyl)butyl]-berberine bromide

9-O-[3-(Phenylamino)propyl]-berberine bromide

JATRORRHIZINE

chelerythrine

1,2-Di(berberine-9-O-yl)ethane dibromide

Papaverine

1,3-Di(berberine-9-O-yl)ethane dibromide

1,4-Di(berberine-9-O-yl)ethane dibromide

1,4-Di(berberine-9-O-yl)ethane dibromide

9-O-[3-(4-Bromo-phenoxyl)butyl]-berberine bromide

9-O-[3-(4-Nitro-phenoxyl)butyl]-berberine bromide

Sanguinarine

Avicine

Pseudopalmatine trifluoroacetate

6,7-Dimethoxy-3-(2-methoxy-phenyl)-quinoline

3-(3,4-Dimethoxy-phenyl)-6,7-dimethoxy-quinoline

6,7-Dimethoxy-3-(4-methoxy-phenyl)-quinoline

6,7-Dimethoxy-3-(3-methoxy-phenyl)-quinoline

NK314

6,7-Dimethoxy-3-pyridin-4-yl-quinoline

6,7-Dimethoxy-3-pyridin-3-yl-quinoline

6,7-Dimethoxy-3-((E)-styryl)-quinoline

6,7-Dimethoxy-3-phenyl-quinoline

6,7-Dimethoxy-3-p-tolyl-quinoline

SALVINORIN B

DEOXY SALVINORIN A

SALVINORIN A

Salvinorin A (ester)

12-EPI-SALVINORIN A

BOLDINE

N-methyllaurotetanine

Predicentrine methiodide

(S)-BULBOCAPNINE

NORBOLDINE

Norisoboldine

N-isopropylnorlitebamine

(R)-(-)-2-methoxy-N-npropylnorapomorphine

2-methoxyapomorphine

GLAUCINE

PUKATEINE

N-propylnorlitebamine

N-allylnorlitebamine

N-butylnorlitebamine

3-Iodoboldine

N-isobutylnorlitebamine

N-isopropylnorlitebamineN-methoiodide

[R-(-)-Apomorphine-2-yl]-(2'-hydroxy-ethyl)ether

2-{[R-(-)-Apomorphine-2'-oxy]ethoxy}-ethanol

N-benzylnorlitebamine

STEPHOLIDINE

(R)-(-)-2-methoxy-11-hydroxyaporphine

(R)-(-)-N-propyl-2-methoxy-11-hydroxynoraporphine

(R)-(-)-N-ethyl-2-methoxy-11-hydroxynoraporphine

(+/-)-nantenine

ORIPAVINE

1,2-Bis-[R-(-)-apomorphine-2'-oxy]ethane

Bis-{[R-(-)-apomorphine-2-oxy]ethyl} ether

Nalorphine

Morphine

ANTIOQUINE

Etorphine

ISOPILINE

Codeine

Ethylmorphine

(S,R)-antioquine hydrochloride

Dihydromorphine

Ro-21-7767

[(R)-(+)-deoxytylophorinidine

COCLAURINE

(R)-(+)-coclaurine

NOR-ROEFRACTINE

ANOLOBINE

(+-)-tetrahydropalmatine

Apomorphine

(S)APOMORPHINE

Tubocurarine

HOMOAROMOLINE

(R,S)-homoaromaline hydrochloride

Dihydrocodeine

Diprenorphine

Buprenorphine + naloxone

[3H]diprenorphine

Apomorphine SL

(R)-(-)-2-Methyl-apomorphine hydrochloride

N-Ethyl-2-methylnorapomorphine hydrochloride

N-Propyl-2-methylnorapomorphine hydrochloride

Naloxegol

Tylophorinidine

Trimetoquinol

THEBAINE

A-69024

[11C]DTBZ

Glaziovine

O-methyldauricine

DIMETHYLGRISABINE

ESCHOLTZINE

NSC-134754

ST-570

Emetine

Tetrabenazine

Deutetrabenazine

Pergularinine

Salvinorin B fluoromethyl ether

2-THIOSALVINORIN B

2-EPI-2-THIOSALVINORIN B

Salvinorin B methylthiomethyl ether

Salvinorin B methoxymethyl ether

Salvinorin B ethoxymethyl ether

2-EPI-2-THIOSALVINORIN A

Salvinorin B isopropoxymethyl ether

Salvinorin B propoxymethyl ether

Salvinorin B butoxymethyl ether

HERKINORIN

Salvinorin B 2-fluoroethoxymethyl ether

Salvinorin B tert-butoxymethyl ether

Salvinorin B 2-methoxyethoxymethyl ether

Salvinorin B 1-ethoxyethyl ether

Salvinorin B 2,2,2-trifluoroethoxymethyl ether

2-methoxy-3,4-methylenedioxybenzophenone

Granulosin

WR-289012

Calphostin C

NSC-87509

FAD-104

3alpha,24-dihydroxyolean-12-en-27-oic acid

24-hydroxyursolic acid

O7-Nitrooxyethyl chrysin

6-iodo-4'-hydroxyflavone

MRS928

MRS923

(+/-)-7-methoxy-2-(4-methoxyphenyl)chroman-4-one

7-methoxy-2-p-tolyl-4H-chromen-4-one

(+/-)-7-methoxy-2-phenylchroman-4-one

2-(4-fluorophenyl)-7-methoxy-4H-chromen-4-one

5-Methoxyflavone

6-Hydroxy-2-(4-hydroxy-benzyl)-chromen-4-one

11-keto-beta-boswellicacid

TRIPTOCALLINE A

Neoandrographolide

Andrographolide

bisandrographolide

Cynarin

ROSMARINIC ACID

Deguelin

Asterric acid

24-ethylcholest-6-ene-3,5-diol

4-ANDROSTENE-3-17-DIONE

NSC-93358

Guggulsterone

methyl p-hydroxybenzoate

4-Hydroxybenzoicacid

4-oxo-4H-chromene-3-carboxylic acid

3-(heptyloxy)benzoic acid

4-Sulfamoyloxy-benzoic acid propyl ester

4-Sulfamoyloxy-benzoic acid butyl ester

VT-1

AR-C70484XX

4-Sulfamoyloxy-benzoic acid heptyl ester

4-Sulfamoyloxy-benzoic acid hexyl ester

4-Sulfamoyloxy-benzoic acid pentyl ester

Bis(6-hydroxybenzo[b]furan-2-yl)methanone

ALBAFURAN A

Mulberrofuran W

eucalyptol

OLEIC ACID

LINOLEIC ACID

Gamma-Homolinolenic acid

(11E)-OCTADEC-11-ENOIC ACID

(E)-5-octadecen-7,9-diynoic acid

(Z)-5-octadecen-7,9-diynoic acid

Icosapent

ISOSCOPOLETIN

SCOPOLETIN

esculetin

HERNIARIN

7-butoxy-2H-chromen-2-one

7-propoxy-2H-chromen-2-one

7,8-dihydroxy-4-phenyl-2H-chromen-2-one

Ethyl 7-methoxy-2-oxo-2H-chromene-3-carboxylate

Geranylcoumarin

7-(benzyloxy)-2H-chromen-2-one

ONO-2506

4-Oxosebacic Acid

Alpha-eudesmol

20S-hydroxycholesterol

ITdU

Patuletin 3-O-beta-D-galactoside

Patuletin 3-O-beta-D-robinobioside

3-[1-ethyl-2-(3-hydroxyphenyl)butyl]phenol

3alpha-Hydroxyurs-12-en-28-oic Acid

Betain anhydrous

[2-(3-Benzyl-3H-indol-1-yl)-ethyl]-dimethyl-amine

NLG8189

AN-1792

PHE-377

Kaempferol-3-O-(2''-O-galloyl)-glucoside

(-)-CATECHINGALLATE

COMBETASTATIN

2-Methoxy-5-(3,4,5-trimethoxy-benzyl)-phenol

3-benzyloxy-4-methoxy-2'-hydroxychalcone

1,2-Bis-(3-methoxy-phenyl)-ethane-1,2-dione

M-2001

PAT-CM-1

3-oxoolean-12-en-27-oic acid

2-(2-Hydroxy-phenyl)-6-methyl-chromen-4-one

OCTYL_GALLATE

Procyanidin B-2 3,3'-di-O-gallate

Levovist

Melanocortin-4 Receptor antagonist

(R,S)-4-phosphonophenylglycine

2-(4-hydroxylphenyl)-3-(3,5-dihydroxylphenyl) propenoic acid (NNU-hdpa)

2beta,3alpha-dihydroxyolean-12-en-28-oic acid

2beta,3alpha-dihydroxyurs-12-en-28-oic acid

2,4-Diamino-butyric acid(GABA)

GALLICACID

2-oxoguaia-1,4(15), 11(13)-trien-12,8beta-olide

2-oxoguaia-1,4,11(13)-trien-12,8alpha-olide

Salvinorin B 2-methoxy-2-propyl ether

3-(4-Hydroxyphenyl)-7-isobutoxychromen-4-one

6,7'-oxybis(2-phenyl-4H-chromen-4-one)

Indol-3-carbinol

Angelicoidenol

cis-Sesquisabinene hydrate

(-)-camphene

(-)-Linalool

(?)-10-Epizonarene

(+)-alpha-phellandrene

(+)-Sabinene

(E)-.beta.-Farnesene

(S)-3alpha-[(S)-1,5-Dimethyl-4-hexenyl]-6-methylenecyclohexene

[4]-Gingerdiacetate

[6]-Gingerdiol 3,5-diacetate

1-(4-hydroxy-3-methoxyphenyl)tetradecane-3,5-diol

1-Dehydro-6-gingerdione

1-Nonanol

3-Methylbutanal

6-Gingerdiol

6-Gingesulfonic acid

6-Shogaol

alpha-Farnesene

alpha-TERPINEOL

beta-Bisabolene

BETA-PHELLANDRENE

BETA-PINENE

beta-Sesquiphellandrene

Citral

Dehydro-10-gingerdione

Dehydrozingerone

Dipentene

DITERPENE II (LACTONE)

DL-Arginine

DL-ASPARTIC ACID

DL-Valine

ent-Zingiberene

Eucalyptol

GAMMA-TERPINENE

GERANIOL

GERANYL ACETATE

Gingerdiol

Gingerdione

Gingerenone A

Gingerenone B

Gingerenone C

Gingerglycolipid A

Gingerglycolipid B

gingerol

glycine

HEPTANE

Hexahydrocurcumin

Isogingerenone B

l-alpha-Curcumene

L-cysteine

l-isoleucine

L-leucine

L-serine

L-threonine

L(-)-Borneol

Methyl-[12]-Gingediol

MYRCENE

nerol

octane

sesquithujene

Vanillylacetone

Phytosterols

(2E,4E)-N-Isobutyl-2,4-decadienamide

2-PHENYLETHANOL

4'-Methoxyacetophenone

6,7-dibromo-4-hydroxy-1H,2H,3H,4H-pyrrolo[1,2-a]pyrazin-1-one

alpha thujene

Aristololactam

BETA-CARYOPHYLLENE

Diaeudesmin

Dihydrocarveol

EICOSANE

Fargesin

guineensine

HENEICOSANE

HEPTADECANE

HEXADECANE

Lignans machilin F

Methyl 3,4,5-trimethoxycinnamate

NONADECANE

OCTADECANE

P-CYMENE

piperine

Piperlongumine

Piperlonguminine

PIPERNONALINE

Piperundecalidine

PLUVIATILOL

TERPINOLENE

TRIACONTANE

1-Methylhexyl acetate

2-HEPTANOL

2-HEPTANONE

2-NONANOL

2-NONANONE

2-Nonyl acetate

Acetyleugenol

Biflorin

Casuarictin

Eugeniin

Eugenin

eugenol

heptan-2-one

Isoeugenitol

naphthalene

Pentagalloyl Glucose

SCHEMBL6761380

Syzyginin A

Syzyginin B

3beta-Hydroxy-20(29)-lupene

Isoflavone glycoside

Xylan,Birch

3-Methylnonacosane

Apigenin 7-glucuronide

BETA-D-XYLOPYRANOSE

Betulin

ethanol

l-ascorbic acid

L-histidine

Lipase

nicotinate

octadeca-9,12-dienoic acid

Octadecanoate

oleic acid

palmitic acid

phenylalanine

Potassium

S-2,6-diaminohexanoic acid

sterol

2-Coumarinate

2,4-Dihydroxycinnamic acid

acyclovir

Arachidic acid

arjungenin

arjunglucoside I

Arjunolic acid

Bellericoside

Chebulagic acid

Daucosterol

DL-Asparagine

Docosanoic acid

ETHYL GALLATE

ferulic acid

Gallic acid

Hyptatic acid A

L-Arabinose

phloroglucinol

Punicalagin

pyrogallol

shikimic acid

TANNIC ACID

Terchebin

Terchebulin

Terminolic acid

TRIACONTANOIC ACID

UNII-I76294A13O

Vanillic acid

11,13-Epoxydehydrocostuslactone

alpha-Cyclocostunolide

.alpha.-Elemene

(+)-germacrene A

1,3-dibutyl-4-methylbenzene

1,8,11,14-Heptadecatetraene

Alantolactone

alpha-Costol

ALPHA-IONONE

ALPHA-SELINENE

beta-Cyclocostunolide

beta-Ionene

BETA-SELINENE

Costic acid

Costol

Costunolide

Dehydrocostus lactone

DIOSGENIN

Friedlein

gamma-Costol

germacra-1(10),4,11(13)-trien-12-ol

I-22,23-Dihydrostigmasterol

Isoalantolactone

Isodehydrocostus lactone

Isozaluzanin C

Picrotoxinum

Saussurea lactone

Saussureamine A

Saussureamine B

Saussureamine C

Taraxasterol

trans-alpha-Bergamotene

1-Heptacosanol

1-Octacosanol

15-Nonacosanone

berberine

Chasmanthin

Columbin

Cordifolide A

Magnoflorine

Palmarin

Tembetarine

Tinosponone

Tinosporinone

xanosporic acid

triterpenoids

apigenin

baicalein

eupatin

luteolin

OLEANOLIC ACID

Queretaroic acid

Scutellarein

Serratagenic acid

Verbenalin

14-Deoxyandrographoside

Dehydroandrographoline

MLS001143515

Stigmasta-5,22-dien-3-ol

14-Deoxy-11,12-didehydroandrographolide

14-deoxyandrographolide

3-O-caffeoyl-D-quinic acid

5-Hydroxy-7,8,2'-trimethoxyflavone 5-glucoside

5-hydroxy-7,8,2',3'-tetramethoxyflavone

andrograpanin

Andrographidine A

Andrographidine C

Andrographidine E

Andrographin

Andrographiside

andropanoside

CARVACROL

CHEMBL479285

Citrostadienol

Deoxyandrographolide

beta-Rotunol

Mustakone

UNII-0V56HXQ8N5

(-)-Rotundone

3-Carene

4-METHOXYBENZOIC ACID

alfa-Cyperone

alpha-rotunol

Arsenic

Aureusidin

beta-Cyperone

Calcium

Copper

Cyperene

Cyperenone

Cyperol

Cyperolone

Cyperotundone

glycerol

Iron

Isocyperol

Isokobusone

Kobusone

linolenic acid

Magnesium

Nickel

quercetin

resmethrin

Scoparone

Sesquiterpene II alcohol diglucoside (Compound 1)

Tetradecanoic acid

Anacyclin

dodeca-2E,4E-dienoic acid isobutylamide

Isobutyramide

Lidocaine hydrochloride

Pellitorine

2,4-dimethyl hexane

2,6-dimethyl heptane

stigmasterol

rutin

Vasicine

vasicinone

vasicinolone

anisotine

vasicoline

Cirsimaritin

oxalacetic acid

THYMOL

Ecdysterone

.alpha.-Amyrin

betaine

choline

Cryptolepine

Hypaphorine

Peganine

Phenethylamine

PSEUDOEPHEDRINE

Vasicinol

[(2R,4R,5S,6R)-3,3,4,5-tetrahydroxy-2-propoxy-6-[[(2S,3S,4S,5R,6R)-3,4,5-trihydroxy-6-(hydroxymethyl)oxan-2-yl]oxymethyl]oxan-4-yl] (Z)-octadec-9-enoate

(4Z)-4,11,11-trimethyl-8-methylidenebicyclo[7.2.0]undec-4-en-7-ol

2,3-Digalloylglucose

AC1L4F91

Chebulinic acid

Inulin from chicory

AC1LAVB6
