# Supplementary material for: Interleukin-10 as Covid-19 biomarker targeting KSK and its analogues: Integrated network pharmacology
Source: PLoS One. 2023 Mar 29;18(3):e0282263. doi: 10.1371/journal.pone.0282263 (PMC10057793; doi:10.1371/journal.pone.0282263)
Supplement: S2 File — (DOCX) [file pone.0282263.s002.docx]

| **CIDs** | **Lipinski #violations** | **Bioavailability Score** | **PAINS #alerts** | **Synthetic Accessibility** |
| --- | --- | --- | --- | --- |
| 5273755 | 0 | 0.6 | 0 | 3.39 |
| 5280666 | 0 | 0.6 | 0 | 3.06 |
| 5326145 | 0 | 0.6 | 0 | 3.12 |
| 5315773 | 0 | 0.6 | 0 | 3.57 |
| 5281654 | 0 | 0.6 | 0 | 3.26 |
| 5281699 | 0 | 0.6 | 0 | 3.26 |
| 5380905 | 0 | 0.6 | 0 | 3.4 |
| 72281 | 0 | 0.6 | 0 | 3.22 |
| 73635 | 0 | 0.6 | 0 | 3.27 |
| 72344 | 0 | 0.6 | 0 | 3.9 |
| 629965 | 0 | 0.6 | 0 | 3.62 |
| 5280442 | 0 | 0.6 | 0 | 2.98 |
| 9948888 | 0 | 0.6 | 0 | 2.44 |
| 5281666 | 0 | 0.6 | 0 | 3.16 |
| 10708015 | 0 | 0.6 | 0 | 2.96 |
| 10286022 | 0 | 0.6 | 0 | 2.86 |
| 5280862 | 0 | 0.6 | 0 | 3.2 |
| 188323 | 0 | 0.6 | 0 | 3.27 |
| 5281703 | 0 | 0.6 | 0 | 3.15 |
| 10568125 | 0 | 0.6 | 0 | 3.56 |
| 44543260 | 0 | 0.6 | 0 | 2.05 |
| 10308075 | 0 | 0.6 | 0 | 1.53 |
| 100801 | 0 | 0.6 | 0 | 2.74 |
| 44542195 | 0 | 0.6 | 0 | 1.98 |
| 247839 | 0 | 0.6 | 0 | 5.15 |
| 5755 | 0 | 0.6 | 0 | 5.23 |
| 31378 | 0 | 0.6 | 0 | 5.25 |
| 31307 | 0 | 0.6 | 0 | 5.46 |
| 6305 | 0 | 0.6 | 0 | 2.09 |
| 9060 | 0 | 0.6 | 0 | 2.09 |
| 676159 | 0 | 0.6 | 0 | 2.29 |
| 6997510 | 0 | 0.6 | 0 | 3.07 |
| 702558 | 0 | 0.6 | 0 | 2.68 |
| 2724998 | 0 | 0.6 | 0 | 1.98 |
| 6931234 | 0 | 0.6 | 0 | 1.98 |
| 67313626 | 0 | 0.6 | 0 | 2.36 |
| 3676 | 0 | 0.6 | 0 | 1.77 |
| 53231 | 0 | 0.6 | 0 | 1.82 |
| 71157 | 0 | 0.6 | 0 | 1.74 |
| 92253 | 0 | 0.6 | 0 | 2.72 |
| 175805 | 0 | 0.6 | 0 | 2.61 |
| 5280863 | 0 | 0.6 | 0 | 3.14 |
| 5281616 | 0 | 0.6 | 0 | 3.12 |
| 5281670 | 0 | 0.6 | 0 | 3.25 |
| 5393152 | 0 | 0.6 | 0 | 3.06 |
| 5280443 | 0 | 0.6 | 0 | 2.96 |
| 5281607 | 0 | 0.6 | 0 | 2.93 |
| 5282073 | 0 | 0.6 | 0 | 2.9 |
| 10084377 | 0 | 0.6 | 0 | 2.94 |
| 122850 | 0 | 0.6 | 0 | 3.42 |
| 10705949 | 0 | 0.6 | 0 | 2.91 |
| 114829 | 0 | 0.6 | 0 | 2.95 |
| 68071 | 0 | 0.6 | 0 | 2.96 |
| 10356745 | 0 | 0.6 | 0 | 3.08 |
| 637308 | 0 | 0.6 | 0 | 2.79 |
| 10913542 | 0 | 0.6 | 0 | 4.39 |
| 12000158 | 0 | 0.6 | 0 | 4.39 |
| 476857 | 0 | 0.6 | 0 | 2.98 |
| 442793 | 0 | 0.6 | 0 | 2.81 |
| 5274976 | 0 | 0.6 | 0 | 2.12 |
| 16756780 | 0 | 0.6 | 0 | 3.51 |
| 6474893 | 0 | 0.6 | 0 | 2.76 |
| 12309636 | 0 | 0.6 | 0 | 3.99 |
| 100154 | 0 | 0.6 | 0 | 3.07 |
| 192827 | 0 | 0.6 | 0 | 3.55 |
| 181681 | 0 | 0.6 | 0 | 4.21 |
| 10040223 | 0 | 0.6 | 0 | 3.88 |
| 10425993 | 0 | 0.6 | 0 | 2.41 |
| 193042 | 0 | 0.6 | 0 | 3.59 |
| 44306276 | 0 | 0.6 | 0 | 2.53 |
| 44306687 | 0 | 0.6 | 0 | 2.59 |
| 13015959 | 0 | 0.6 | 0 | 3.18 |
| 44306292 | 0 | 0.6 | 0 | 2.49 |
| 10356352 | 0 | 0.6 | 0 | 2.02 |
| 44575398 | 0 | 0.6 | 0 | 4.3 |
| 91510 | 0 | 0.6 | 0 | 3.7 |
| 3013841 | 0 | 0.6 | 0 | 4.03 |
| 44418728 | 0 | 0.6 | 0 | 3.61 |
| 44418722 | 0 | 0.6 | 0 | 3.4 |
| 44418723 | 0 | 0.6 | 0 | 3.62 |
| 44418729 | 0 | 0.6 | 0 | 3.51 |
| 44418717 | 0 | 0.6 | 0 | 3.15 |
| 16066665 | 0 | 0.6 | 0 | 3.61 |
| 10469827 | 0 | 0.6 | 0 | 3.06 |
| 73571 | 0 | 0.6 | 0 | 3.11 |
| 160481 | 0 | 0.6 | 0 | 3.11 |
| 46914767 | 0 | 0.6 | 0 | 3.22 |
| 637048 | 0 | 0.6 | 0 | 3.08 |
| 3764 | 0 | 0.6 | 0 | 2.86 |
| 5280373 | 0 | 0.6 | 0 | 2.89 |
| 5280378 | 0 | 0.6 | 0 | 2.81 |
| 5281804 | 0 | 0.6 | 0 | 2.96 |
| 11414445 | 0 | 0.6 | 0 | 1.62 |
| 44428616 | 0 | 0.6 | 0 | 2.85 |
| 5280961 | 0 | 0.6 | 0 | 2.87 |
| 5281708 | 0 | 0.6 | 0 | 2.79 |
| 134497 | 0 | 0.6 | 0 | 2.05 |
| 11622683 | 0 | 0.6 | 0 | 2.3 |
| 185915 | 0 | 0.6 | 0 | 1.7 |
| 7020128 | 0 | 0.6 | 0 | 2.16 |
| 60818 | 0 | 0.6 | 0 | 5.09 |
| 72323 | 0 | 0.6 | 0 | 3.06 |
| 4680 | 0 | 0.6 | 0 | 2.62 |
| 10085862 | 0 | 0.6 | 0 | 2.5 |
| 10336427 | 0 | 0.6 | 0 | 2.62 |
| 9882618 | 0 | 0.6 | 0 | 2.33 |
| 10447210 | 0 | 0.6 | 0 | 2.47 |
| 10061448 | 0 | 0.6 | 0 | 2.09 |
| 10445611 | 0 | 0.6 | 0 | 2.17 |
| 10017166 | 0 | 0.6 | 0 | 2.38 |
| 10422972 | 0 | 0.6 | 0 | 2.17 |
| 10401378 | 0 | 0.6 | 0 | 2.3 |
| 10154 | 0 | 0.6 | 0 | 3.58 |
| 16573 | 0 | 0.6 | 0 | 3.69 |
| 12441 | 0 | 0.6 | 0 | 3.77 |
| 22179 | 0 | 0.6 | 0 | 3.46 |
| 14539911 | 0 | 0.6 | 0 | 3.43 |
| 16754 | 0 | 0.6 | 0 | 3.8 |
| 442340 | 0 | 0.6 | 0 | 3.63 |
| 6917970 | 0 | 0.6 | 0 | 3.35 |
| 24766199 | 0 | 0.6 | 0 | 3.39 |
| 24800688 | 0 | 0.6 | 0 | 3.61 |
| 5462306 | 0 | 0.6 | 0 | 4.88 |
| 5284595 | 0 | 0.6 | 0 | 4.93 |
| 5288826 | 0 | 0.6 | 0 | 4.78 |
| 13891896 | 0 | 0.6 | 0 | 3.43 |
| 5284371 | 0 | 0.6 | 0 | 4.89 |
| 5359271 | 0 | 0.6 | 0 | 5 |
| 5359421 | 0 | 0.6 | 0 | 4.56 |
| 160487 | 0 | 0.6 | 0 | 2.78 |
| 440989 | 0 | 0.6 | 0 | 2.78 |
| 7055406 | 0 | 0.6 | 0 | 2.9 |
| 6000 | 1 | 0.6 | 0 | 6.92 |
| 99620 | 1 | 0.6 | 0 | 6.89 |
| 9851833 | 1 | 0.6 | 0 | 6.89 |
| 5284543 | 0 | 0.6 | 0 | 4.67 |
| 443408 | 0 | 0.6 | 0 | 5.94 |
| 6858272 | 0 | 0.6 | 0 | 5.94 |
| 161749 | 0 | 0.6 | 0 | 3.5 |
| 5324289 | 0 | 0.6 | 0 | 4.98 |
| 200521 | 1 | 0.6 | 0 | 5.32 |
| 14137099 | 1 | 0.6 | 0 | 5.32 |
| 12304178 | 0 | 0.6 | 0 | 3.43 |
| 3246465 | 0 | 0.6 | 0 | 4.8 |
| 10219 | 0 | 0.6 | 0 | 4.87 |
| 73437646 | 0 | 0.6 | 0 | 3.66 |
| 264751 | 0 | 0.6 | 0 | 3.61 |
| 44456305 | 0 | 0.6 | 0 | 5.35 |
| 17747979 | 0 | 0.6 | 0 | 5.21 |
| 44425463 | 0 | 0.6 | 0 | 5.21 |
| 44456307 | 0 | 0.6 | 0 | 5.68 |
| 44456192 | 0 | 0.6 | 0 | 5.6 |
| 44425462 | 0 | 0.6 | 0 | 5.55 |
| 44456377 | 0 | 0.6 | 0 | 5.84 |
| 44456420 | 0 | 0.6 | 0 | 5.84 |
| 44456421 | 0 | 0.6 | 0 | 5.96 |
| 11431898 | 0 | 0.6 | 0 | 5.55 |
| 44456375 | 0 | 0.6 | 0 | 5.64 |
| 44456378 | 0 | 0.6 | 0 | 5.96 |
| 44456347 | 0 | 0.6 | 0 | 5.9 |
| 44456346 | 1 | 0.6 | 0 | 5.62 |
| 11999967 | 0 | 0.6 | 0 | 2.64 |
| 10704719 | 0 | 0.6 | 0 | 3 |
| 44419435 | 0 | 0.6 | 0 | 3.18 |
| 5320418 | 0 | 0.6 | 0 | 3.95 |
| 44568920 | 1 | 0.6 | 0 | 6.36 |
| 44590927 | 0 | 0.6 | 0 | 3.54 |
| 117900 | 0 | 0.6 | 0 | 3.45 |
| 631105 | 0 | 0.6 | 0 | 3.87 |
| 930709 | 0 | 0.6 | 0 | 3.1 |
| 10378415 | 0 | 0.6 | 0 | 2.95 |
| 94525 | 0 | 0.6 | 0 | 2.97 |
| 10539813 | 0 | 0.6 | 0 | 2.88 |
| 9847548 | 1 | 0.9 | 0 | 6.24 |
| 44559634 | 0 | 0.6 | 0 | 6.01 |
| 9848024 | 0 | 0.6 | 0 | 6.36 |
| 5318517 | 0 | 0.6 | 0 | 5.06 |
| 107935 | 0 | 0.6 | 0 | 4.67 |
| 3080568 | 0 | 0.6 | 0 | 2.93 |
| 6128 | 0 | 0.6 | 0 | 4.64 |
| 6450278 | 0 | 0.6 | 0 | 4.79 |
| 7456 | 0 | 0.6 | 0 | 1.02 |
| 135 | 0 | 0.9 | 0 | 1 |
| 181620 | 0 | 0.9 | 0 | 2.43 |
| 14368760 | 0 | 0.9 | 0 | 2.12 |
| 10858185 | 0 | 0.6 | 0 | 2.29 |
| 10880277 | 0 | 0.6 | 0 | 2.4 |
| 9881384 | 0 | 0.6 | 0 | 2.4 |
| 9796948 | 0 | 0.6 | 0 | 2.76 |
| 9879424 | 0 | 0.6 | 0 | 2.64 |
| 11822292 | 0 | 0.6 | 0 | 2.52 |
| 13393486 | 0 | 0.6 | 0 | 2.76 |
| 5281297 | 0 | 0.6 | 0 | 3.9 |
| 45273151 | 1 | 0.6 | 0 | 4.4 |
| 2758 | 0 | 0.6 | 0 | 3.65 |
| 445639 | 1 | 0.9 | 0 | 3.07 |
| 5280450 | 1 | 0.9 | 0 | 3.07 |
| 5280581 | 1 | 0.9 | 0 | 3.07 |
| 5281127 | 1 | 0.9 | 0 | 3.07 |
| 9543636 | 1 | 0.9 | 0 | 4.02 |
| 9543637 | 1 | 0.9 | 0 | 4.02 |
| 446284 | 1 | 0.9 | 0 | 4.02 |
| 69894 | 0 | 0.6 | 0 | 2.65 |
| 5280460 | 0 | 0.6 | 0 | 2.62 |
| 10748 | 0 | 0.6 | 0 | 2.59 |
| 156219 | 0 | 0.6 | 0 | 2.77 |
| 1631814 | 0 | 0.6 | 0 | 2.67 |
| 914298 | 0 | 0.6 | 0 | 2.89 |
| 44429171 | 0 | 0.6 | 0 | 3.52 |
| 910172 | 0 | 0.6 | 0 | 2.75 |
| 208925 | 0 | 0.9 | 0 | 2.22 |
| 1755 | 0 | 0.6 | 0 | 1.89 |
| 92762 | 0 | 0.6 | 0 | 4.08 |
| 440966 | 1 | 0.6 | 0 | 3.5 |
| 443158 | 0 | 0.6 | 0 | 2.74 |
| 10954686 | 1 | 0.6 | 0 | 4.43 |
| 443160 | 0 | 0.6 | 0 | 4.15 |
| 10887971 | 1 | 0.6 | 0 | 2.87 |
| 10407 | 1 | 0.6 | 0 | 3.42 |
| 12315493 | 1 | 0.6 | 0 | 4.42 |
| 5317587 | 0 | 0.6 | 0 | 3.7 |
| 9796015 | 0 | 0.6 | 0 | 2.83 |
| 8914 | 0 | 0.6 | 0 | 1.54 |
| 11552 | 0 | 0.6 | 0 | 1 |
| 11369949 | 0 | 0.6 | 0 | 3.14 |
| 5281794 | 0 | 0.6 | 0 | 2.51 |
| 5281516 | 1 | 0.6 | 0 | 3.72 |
| 10104370 | 1 | 0.6 | 0 | 3.9 |
| 14896 | 1 | 0.6 | 0 | 3.73 |
| 12315492 | 1 | 0.6 | 0 | 4.42 |
| 638011 | 0 | 0.6 | 0 | 2.49 |
| 22608831 | 0 | 0.9 | 0 | 3.48 |
| 11127403 | 1 | 0.6 | 0 | 4.81 |
| 7461 | 0 | 0.6 | 0 | 3.11 |
| 637566 | 0 | 0.6 | 0 | 2.58 |
| 1549026 | 0 | 0.6 | 0 | 2.72 |
| 162952 | 0 | 0.6 | 0 | 2.23 |
| 5281775 | 0 | 0.6 | 0 | 2.74 |
| 5317592 | 0 | 0.6 | 0 | 2.99 |
| 5317593 | 0 | 0.6 | 0 | 2.59 |
| 750 | 0 | 0.6 | 0 | 1 |
| 8900 | 0 | 0.6 | 0 | 1.33 |
| 5318568 | 0 | 0.6 | 0 | 2.99 |
| 442360 | 1 | 0.6 | 0 | 2.31 |
| 5862 | 0 | 0.6 | 0 | 1.75 |
| 6288 | 0 | 0.6 | 0 | 1.79 |
| 1201518 | 0 | 0.6 | 0 | 3.43 |
| 31253 | 0 | 0.6 | 0 | 2.85 |
| 643820 | 0 | 0.6 | 0 | 2.58 |
| 356 | 1 | 0.6 | 0 | 1.42 |
| 53359349 | 1 | 0.6 | 0 | 4.61 |
| 31211 | 0 | 0.6 | 0 | 1.52 |
| 11368078 | 1 | 0.6 | 0 | 6.3 |
| 6054 | 0 | 0.6 | 0 | 1 |
| 7476 | 0 | 0.6 | 0 | 1 |
| 96710 | 0 | 0.6 | 0 | 2.56 |
| 6992053 | 0 | 0.6 | 0 | 4.22 |
| 10926754 | 0 | 0.6 | 0 | 4.3 |
| 6442405 | 0 | 0.6 | 0 | 3.8 |
| 12398 | 1 | 0.6 | 0 | 2.38 |
| 11006 | 1 | 0.6 | 0 | 2.26 |
| 13844301 | 0 | 0.6 | 0 | 4.13 |
| 735846 | 0 | 0.6 | 0 | 2.47 |
| 11635 | 1 | 0.6 | 0 | 2.49 |
| 7463 | 1 | 0.6 | 0 | 1 |
| 637858 | 0 | 0.6 | 0 | 3.18 |
| 5320621 | 0 | 0.6 | 0 | 2.98 |
| 44453654 | 0 | 0.6 | 0 | 3.53 |
| 70695727 | 0 | 0.6 | 0 | 4.19 |
| 11463 | 0 | 0.6 | 0 | 2.98 |
| 8051 | 0 | 0.6 | 0 | 1.33 |
| 13187 | 0 | 0.6 | 0 | 1.52 |
| 7136 | 0 | 0.6 | 0 | 1.94 |
| 441959 | 1 | 0.6 | 0 | 4.61 |
| 10189 | 0 | 0.6 | 0 | 2.62 |
| 5318562 | 0 | 0.6 | 0 | 2.73 |
| 1369 | 0 | 0.9 | 0 | 2.95 |
| 10360205 | 0 | 0.6 | 0 | 4.48 |
| 73337 | 0 | 0.6 | 0 | 3.78 |
| 167718 | 0 | 0.6 | 0 | 3.21 |
| 15215479 | 0 | 0.6 | 0 | 4.91 |
| 5317287 | 0 | 0.6 | 0 | 3.59 |
| 5708351 | 0 | 0.6 | 0 | 5.29 |
| 11624161 | 0 | 0.6 | 0 | 4.82 |
| 5319878 | 0 | 0.6 | 0 | 3.67 |
| 5318506 | 0 | 0.6 | 0 | 3.52 |
| 21679042 | 0 | 0.6 | 0 | 4.95 |
| 158720 | 0 | 0.6 | 0 | 2.76 |
| 82143 | 0 | 0.6 | 0 | 1.71 |
| 442934 | 0 | 0.6 | 0 | 3.35 |
| 76 | 0 | 0.6 | 0 | 4.66 |
| 53956 | 0 | 0.6 | 0 | 2.06 |
| 372869 | 0 | 0.6 | 0 | 2.4 |
| 405012 | 0 | 0.6 | 0 | 2.29 |
| 11032345 | 0 | 0.6 | 0 | 2.96 |
| 44303123 | 0 | 0.6 | 0 | 4.15 |
| 44393544 | 0 | 0.6 | 0 | 3.82 |
| 6314 | 0 | 0.6 | 0 | 1.86 |
| 67249 | 0 | 0.6 | 0 | 3.06 |
| 261365 | 0 | 0.6 | 0 | 4.74 |
| 442935 | 0 | 0.6 | 0 | 2.75 |
| 9884487 | 0 | 0.6 | 0 | 2.11 |
| 73755103 | 0 | 0.6 | 0 | 4 |
