# Supplementary material for: Interleukin-10 as Covid-19 biomarker targeting KSK and its analogues: Integrated network pharmacology
Source: PLoS One. 2023 Mar 29;18(3):e0282263. doi: 10.1371/journal.pone.0282263 (PMC10057793; doi:10.1371/journal.pone.0282263)
Supplement: S3 File — (DOCX) [file pone.0282263.s003.docx]

NR1H3

NR1H2

GPR183

NR1H4

RORC

PCYT1B

RORA

ESR1

ESR2

AChE

BCHE

LSS

ESRRA

TK1

TOP2

SLC6A9

AR

VDR

HMGCR

PTPN1

NR1I3

HSD11B1

PLA2G1B

ADORA3

PTGS2

XDH

TUB

TUBB

CYP1B1

SELL

GLO1

SELP

PREP

TNF

CYP19A1

TERT

AKR1B1

ODC1

HIF1AN

DGAT1

PDE5A

ADORA1

CDK6

FASN

TYR

USPL1

PIK3CG

CDK5

IL4R

KDR

BACE1

ABCA12

MCL1

ALOX12

ALOX15

PDE3A

PDE4A

PDE4B

CSNK2A1

ALOX5

ADORA2A

SLC22A12

GRM7

ADORA2B

CYP2D6

ACE

AKR1C3

CACNA1B

ALDH2

IL5

CYP3A4

SLC5A1

SLCO1A2

SQLE

SLC5A2

HTR2C

TRPV3

CA2

CNR1

PTGS1

TRPM4

IDO1

KIF11

NR3C1

NR3C2

KCNA3

HTR6

NQO2

MAOA

HCAR3

SLC6A14

SLC36A2

TPH2

TPH1

SLC36A1

STAT3

ECE1

EDNRB

PPARG

TACR1

DNMT1

DNMT3B

TAAR1

HTR2A

MAOB

ADRA1B

DRD2

HRH1

SLC6A2

ANPEP

HTR1A

HTR1D

GRIN1

GRIN2B

AOC3

HTR2B

HTR7

PRKAB1

NOS2

PLK1

APP

SLC6A3

REN

INS

ICAM1

CNR2

ALDH3A2

SLC1A2

SLC1A6

GRIK1

CXCL10

SCN11A

TRPV1

RFWD2

XK

SCN10A

SCN5A

KCNA5

SLC29A1

SCN3A

TRPM8

FNTB

XPO1

CA1

CA4

CA9

CA6

CA12

CA14

PMVK

TRPA1

TGM2

SP1

MMP13

MMP9

ABAT

ALDH5A1

AOX1

JAK2

PTAFR

GABRG3

GJE1

GJB7

GJB6

GJB1

GJC1

GJA9

GJA10

GJA1

GJA3

GJA4

GJA5

GJA8

GJB2

GJB3

GJB4

GJB5

GJD2

GJD3

GJD4

GJC2

GJC3

FAAH

NOS1

NOS3

GPRC6A

AKT1S1

CES1

HSPA4

HSPA8

GLA

CRYBB1

CDK2

MGAM

TLR2

ATM

THRA

THRB

STS

ELANE

HPD

TUBB4B

SERPINE1

CYSLTR1

LTB4R

LTB4R2

GRM2

GABRA1

GABRA2

GABRA3

GABRA5

GABRB2

GABRG2

ESRRB

ESRRG

PPARD

CAPN2

EGFR

LIPE

PRKCD

PRKCA

TRPC5

PTGDR

PTGER1

PTGER2

PTGER3

F2

F10

PRKCB

MAPKAP1

SMAD3

SYK

IGFBP3

GCK

AMY2A

ADAM33

SERPINC1

APOH

CTSD

CTLA4

DNASE1

CGA

SERPIND1

IGF1R

IL6R

LCT

LTF

CD209

RTN4R

RHO

CD2

FHIT

LGALS1

HDC

HCAR2

FFAR1

FFAR4

TOP1

SCD

FABP4

FABP5

HNF4A

LY96

TLR4

GPR84

CALM2

FKBP1A

RCVRN

ABL1

SLC22A8

MC4R

HMHB1

RXRA

PPARA

CACNA2D1

CTSB

PAH

SLC1A1

PIN1

S1PR1

GRM8

SLC7A5

HNF1A

NOD1

NR1I2

MIF

CASP8

HSD11B2

KCNH2

CXCR3

SLC6A1

SLC6A11

AKT1

HCAR1

MMP2

SLC37A4

CACNA1A

NRAS

P2RX7

PRKCE

PDE4D

PDGFRA

PDGFRB

TOP2A

OPRK1

OPRD1

OPN4

CHRNA4

CHRNB2

HTR1B

HTR5A

ADRA1A

ADRA1D

ADRA2A

ADRA2B

ADRA2C

ADRB1

HRH2

SLC6A4

POMC

JUN

CHRNA2

CALY

PDF

ADRB3

SLC18A2

EDNRA

GABPA

KIAA0101

TRPV4

SRSF10

AURKB

HSD3B1

FLT3

FABP1

CPOX

COMT

S100B

ALAD

A2M

ABO

ACE2

ADAM17

ADAMTS13

ADIPOQ

ADM

AGER

AGT

AGTR1

AGTR2

ALB

ANGPT2

ANXA2

AP3B1

APOE

APOL1

ARNTL

ATF4

AVP

BCL2

BDH1

BECN1

BSG

BTK

C4BPA

C4BPB

C5

C5AR1

C9orf72

CALCA

CAT

CCL17

CCL2

CCL20

CCL27

CCL3

CCL4

CCL7

CCL8

CCR1

CCR5

GPR29

CCR9

CD14

CD163

CD4

CD8A

CD99

CHI3L1

CHRFAM7A

CLEC4M

CPA3

CRP

CSF1

CSF2

CSF3

CST3

CTSL

CXCL16

CXCL2

CXCL8

CXCR6

DEFA1

DPP4

EDN1

ELAVL2

ENPEP

ENPP2

EPO

ERG

ERVFRD-1

ERVW-1

ESM1

F2RL1

F2RL3

F3

FABP2

FAS

FCGR1A

FCGR3A

FLT1

FNDC5

FPR1

FTH1

FTL

FURIN

G6PD

GC

GDF15

GFAP

GSTM1

GZMA

GZMB

HAVCR2

HIF1A

HLA-A

HLA-B

HLA-C

HLA-DRB1

HLA-G

HMGB1

HMOX1

HSPA5

IFIH1

IFITM3

IFNA1

IFNA2

IFNAR2

IFNB1

IFNG

IFNL1

IFNL2

IL10

IL10RB

IL13

IL17A

IL18

IL1B

IL1R1

IL2

IL22

IL2RA

IL2RB

IL2RG

IL33

IL37

IL4

IL6

IL7

IL9

ITGAV

ITK

JAK1

KLF2

KLRC2

KNG1

LAG3

LCN2

LDHA

LEP

LGALS3

LGALS9

LPA

LZTFL1

MAF

MAFB

ENSP00000485396

MAS1

MB

MBL2

MIR29A

MMP1

MMP3

MMP8

MPO

MTHFR

MTOR

MUC1

MUC5AC

NCAM1

NFE2L2

NLRP3

NPPB

NRP1

NRP2

OAS1

OLR1

P2RY14

PAFAH1B1

PARP14

PER3

PF4

PLA2G2A

PLA2G7

PLAUR

PLG

PNPLA3

PRF1

PTGDR2

PTX3

RBM15

MINA

S100A12

S100A8

S100A9

SAA1

SDC1

SDCBP

SELE

SERPINA1

SERPINF2

SERPING1

SFTPB

SFTPD

SIGLEC1

SIGMAR1

SIRT1

SIRT3

SLC6A19

SLC6A20

SLCO4C1

SMN1

SOCS1

SOCS3

SOD3

SPP1

SREBF2

STAT1

TMEM173

TAS2R1

TAS2R38

TEK

TGFB1

THBS1

THBS3

THPO

TLR3

TLR7

TMPRSS2

TNFRSF13C

TNFRSF1B

TNFSF14

TNNI1

TNNI3

TNNT2

TREM1

TREM2

TTR

UBE2L6

UNC13D

VKORC1

VWF

ZC3HAV1
