# Supplementary material for: Interleukin-10 as Covid-19 biomarker targeting KSK and its analogues: Integrated network pharmacology
Source: PLoS One. 2023 Mar 29;18(3):e0282263. doi: 10.1371/journal.pone.0282263 (PMC10057793; doi:10.1371/journal.pone.0282263)
Supplement: S4 File — (DOCX) [file pone.0282263.s004.docx]

Functional Annotation – **Gene Ontology descriptions** and the number of target genes involved in each of the enriched GO terms.

| **GO** | **Term** | **Genes** | **Count** |
| --- | --- | --- | --- |
| **Biological Process** | cytokine-mediated signaling pathway | IL10, IL4, IL6, CD4, CXCL8, CSF2, IL1B, TNF, IL17A | 9 |
|  | immune response | IL10, IL4, CD4, CXCL8, CSF2, IL1B, TNF, IL17A | 8 |
|  | cellular response to lipopolysaccharide | IL10, IL6, CXCL8, CSF2, IL1B, TNF | 6 |
|  | positive regulation of tyrosine phosphorylation of STAT protein | IL4, IL6, CSF2, IFNG, TNF | 5 |
|  | positive regulation of gene expression | IL4, IL6, CXCL8, CSF2, IFNG, IL1B, TNF | 7 |
|  | positive regulation of interleukin-6 production | IL6, IFNG, IL1B, TNF, IL17A | 5 |
|  | positive regulation of calcidiol 1-monooxygenase activity | IFNG, IL1B, TNF | 3 |
|  | positive regulation of interleukin-1 beta production | IL6, IFNG, TNF, IL17A | 4 |
|  | positive regulation of T cell proliferation | IL4, IL6, CD4, IL1B | 4 |
|  | regulation of insulin secretion | IL6, IFNG, IL1B, TNF | 4 |
|  | vascular endothelial growth factor production | IL6, IL1B, TNF | 3 |
|  | positive regulation of interleukin-23 production | CSF2, IFNG, IL17A | 3 |
|  | positive regulation of transcription, DNA-templated | IL10, IL4, IL6, CD4, IL1B, TNF | 6 |
|  | positive regulation of MHC class II biosynthetic process | IL10, IL4, IFNG | 3 |
|  | positive regulation of sequence-specific DNA binding transcription factor activity | IL10, IL6, IL1B, TNF | 4 |
|  | inflammatory response | IL6, CXCL8, IL1B, TNF, IL17A | 5 |
| **Cellular Component** | extracellular space | IL10, IL4, IL6, CXCL8, CSF2, IFNG, IL1B, TNF, IL17A | 9 |
|  | extracellular region | IL10, IL4, IL6, CXCL8, CSF2, IFNG, IL1B, TNF, IL17A | 9 |
|  | external side of plasma membrane | CD4, TNF, IL17A | 3 |
| **Molecular Function** | cytokine activity | IL10, IL4, IL6, CSF2, IFNG, IL1B, TNF, IL17A | 8 |
|  | growth factor activity | IL10, IL4, IL6, CSF2 | 4 |
|  | protein binding | IL10, IL4, IL6, CD4, CXCL8, CSF2, IFNG, IL1B, TNF, IL17A | 10 |

Functional Annotation – **Pathway analysis** (KEGG and Reactome) and **Diseases related** to the given hub genes.

| **Pathway** | **Term** | **Genes** | **Count** |
| --- | --- | --- | --- |
| **KEGG Pathway** | Cytokine-cytokine receptor interaction | IL10, IL4, IL6, CD4, CXCL8, CSF2, IFNG, IL1B, TNF, IL17A | 10 |
|  | IL-17 signaling pathway | IL4, IL6, CXCL8, CSF2, IFNG, IL1B, TNF, IL17A | 8 |
|  | Inflammatory bowel disease | IL10, IL4, IL6, IFNG, IL1B, TNF, IL17A | 7 |
|  | Rheumatoid arthritis | IL6, CXCL8, CSF2, IFNG, IL1B, TNF, IL17A | 7 |
|  | Amoebiasis | IL10, IL6, CXCL8, CSF2, IFNG, IL1B, TNF | 7 |
|  | Malaria | IL10, IL6, CXCL8, IFNG, IL1B, TNF | 6 |
|  | Hematopoietic cell lineage | IL4, IL6, CD4, CSF2, IL1B, TNF | 6 |
|  | Chagas disease | IL10, IL6, CXCL8, IFNG, IL1B, TNF | 6 |
|  | T cell receptor signaling pathway | IL10, IL4, CD4, CSF2, IFNG, TNF | 6 |
|  | African trypanosomiasis | IL10, IL6, IFNG, IL1B, TNF | 5 |
| **Reactome** | Signaling by Interleukins | IL10, IL4, IL6, CD4, CXCL8, CSF2, IFNG, IL1B, TNF, IL17A | 10 |
|  | Cytokine Signaling in Immune system | IL10, IL4, IL6, CD4, CXCL8, CSF2, IFNG, IL1B, TNF, IL17A | 10 |
|  | Interleukin-4 and Interleukin-13 signaling | IL10, IL4, IL6, CXCL8, IL1B, TNF, IL17A | 7 |
|  | Interleukin-10 signaling | IL10, IL6, CXCL8, CSF2, IL1B, TNF | 6 |
|  | Immune System | IL10, IL4, IL6, CD4, CXCL8, CSF2, IFNG, IL1B, TNF, IL17A | 10 |
|  | CD163 mediating an anti-inflammatory response | IL10, IL6 | 2 |
|  | Leishmania infection | IL10, IL6, IL1B | 3 |
|  | Gene and protein expression by JAK-STAT signaling after Interleukin-12 stimulation | IL10, IFNG | 2 |
|  | Infectious disease | IL10, IL6, CD4, IL1B | 4 |
|  | Interleukin-12 signaling | IL10, IFNG | 2 |
|  | Interleukin-12 family signaling | IL10, IFNG | 2 |
|  | Senescence-Associated Secretory Phenotype (SASP) | IL6, CXCL8 | 2 |
| **DISGENET** | Leishmaniasis, Visceral | IL10, IL6, CXCL8, CSF2, IFNG, IL1B, TNF | 7 |
|  | Leishmaniasis, Cutaneous | IL10, IL4, CXCL8, IFNG, IL1B, TNF | 6 |
|  | Urban cutaneous leishmaniasis | IL10, IL4, CXCL8, IFNG, IL1B, TNF | 6 |
|  | Leishmaniasis, New World | IL10, IL4, CXCL8, IFNG, IL1B, TNF | 6 |
|  | Colitis | IL10, IL4, IL6, IFNG, IL1B, TNF, IL17A | 7 |
|  | Arthritis, Experimental | IL10, IL4, IL6, IFNG, IL1B, TNF, IL17A | 7 |
|  | Arthritis, Adjuvant-Induced | IL10, IL4, IL6, IFNG, IL1B, TNF, IL17A | 7 |
|  | Arthritis, Collagen-Induced | IL10, IL4, IL6, IFNG, IL1B, TNF, IL17A | 7 |
|  | Experimental Lung Inflammation | IL4, IL6, CSF2, IFNG, IL1B, TNF, IL17A | 7 |
|  | Pneumonitis | IL4, IL6, CSF2, IFNG, IL1B, TNF, IL17A | 7 |
|  | Pneumonia | IL4, IL6, CSF2, IFNG, IL1B, TNF, IL17A | 7 |
|  | Lobar Pneumonia | IL4, IL6, CSF2, IFNG, IL1B, TNF, IL17A | 7 |
|  | Inflammation | IL10, IL6, CXCL8, CSF2, IFNG, IL1B, TNF, IL17A | 8 |
|  | Bright Disease | IL10, IL6, CXCL8, IFNG, IL1B, TNF | 6 |
|  | Glomerulonephritis | IL10, IL6, CXCL8, IFNG, IL1B, TNF | 6 |
|  | Allergic Reaction | IL4, IL6, IFNG, IL1B, TNF, IL17A | 6 |
|  | Hypersensitivity | IL4, IL6, IFNG, IL1B, TNF, IL17A | 6 |
|  | Rheumatoid Arthritis | IL10, IL6, CXCL8, CSF2, IFNG, IL1B, TNF | 7 |
|  | Alveolitis, Fibrosing | IL4, IL6, CXCL8, CSF2, IL1B, TNF | 6 |
|  | Pulmonary Fibrosis | IL4, IL6, CXCL8, CSF2, IL1B, TNF | 6 |
|  | Fever | IL6, CXCL8, IFNG, IL1B, TNF | 5 |
