# Supplementary material for: Interleukin-10 as Covid-19 biomarker targeting KSK and its analogues: Integrated network pharmacology
Source: PLoS One. 2023 Mar 29;18(3):e0282263. doi: 10.1371/journal.pone.0282263 (PMC10057793; doi:10.1371/journal.pone.0282263)
Supplement: S5 File — (DOCX) [file pone.0282263.s005.docx]

Hydrophobic Interactions of top nine compounds.

| **Index** | **Residue** | **AA** | **Distance** | **Ligand Atom** | **Protein Atom** | **Protein-Ligand complex** |
| --- | --- | --- | --- | --- | --- | --- |
| 1 | 4A | TYR | 3.95 | 36 | 95 | IFNG_A_6000  (Tubocurarine) |
| 2 | 8A | ALA | 3.65 | 7 | 136 |  |
| 3 | 11A | LEU | 3.9 | 33 | 163 |  |
| 4 | 29A | PHE | 3.79 | 18 | 336 |  |
| 5 | 30A | LEU | 3.72 | 17 | 348 |  |
| 6 | 57A | PHE | 3.83 | 8 | 638 |  |
| 7 | 73A | ILE | 3.98 | 6 | 802 |  |
| 1 | 8A | ALA | 3.53 | 19 | 135 | IFNG_A_99620  (Homoaromoline) |
| 2 | 12A | LYS | 3.88 | 29 | 171 |  |
| 3 | 12A | LYS | 3.79 | 26 | 172 |  |
| 4 | 17A | ALA | 3.99 | 26 | 234 |  |
| 5 | 53A | TYR | 3.89 | 33 | 588 |  |
| 6 | 53A | TYR | 3.76 | 32 | 585 |  |
| 7 | 53A | TYR | 3.51 | 21 | 587 |  |
| 8 | 57A | PHE | 3.61 | 21 | 637 |  |
| 1 | 4A | TYR | 3.55 | 5 | 80 | IFNG_A_44559634  (Triptocalline A) |
| 2 | 8A | ALA | 3.54 | 9 | 123 |  |
| 3 | 11A | LEU | 3.68 | 14 | 152 |  |
| 4 | 17A | ALA | 3.77 | 18 | 222 |  |
| 5 | 53A | TYR | 3.93 | 8 | 576 |  |
| 6 | 53A | TYR | 3.93 | 13 | 575 |  |
| 7 | 73A | ILE | 3.73 | 4 | 789 |  |
| 8 | 77A | MET | 3.4 | 4 | 828 |  |
| 1 | 4A | TYR | 3.48 | 10 | 83 | IFNG_A_44568920  (24-Hydroxyursolic acid) |
| 2 | 49A | ILE | 3.61 | 21 | 539 |  |
| 3 | 73A | ILE | 3.99 | 10 | 792 |  |
| 1 | 60A | TYR | 3.77 | 6 | 605 | IL4_13393486  (Bis(6-hydroxybenzo[b]furan-2-yl)methanone) |
| 2 | 88A | LYS | 3.99 | 5 | 909 |  |
| 3 | 91A | ASP | 3.93 | 6 | 948 |  |
| 4 | 92A | ARG | 3.72 | 3 | 957 |  |
| 1 | 30A | PHE | 3.54 | 15 | 229 | IL10_6450278  (Guggulsterone) |
| 2 | 30A | PHE | 3.49 | 11 | 227 |  |
| 3 | 65A | LEU | 3.65 | 4 | 580 |  |
| 4 | 68A | MET | 3.73 | 4 | 605 |  |
| 5 | 69A | ILE | 3.46 | 4 | 615 |  |
| 6 | 72A | TYR | 3.38 | 9 | 651 |  |
| 7 | 72A | TYR | 3.69 | 20 | 652 |  |
| 8 | 72A | TYR | 3.76 | 8 | 649 |  |
| 9 | 72A | TYR | 3.56 | 7 | 648 |  |
| 10 | 94A | LEU | 3.64 | 13 | 861 |  |
| 11 | 98A | LEU | 3.41 | 7 | 896 |  |
| 1 | 37A | PHE | 3.9 | 7 | 329 | IL10_9851833  ((R,S)-homoaromaline hydrochloride) |
| 2 | 37A | PHE | 3.71 | 14 | 331 |  |
| 3 | 41A | ASP | 3.58 | 5 | 373 |  |
| 4 | 46A | LEU | 3.83 | 29 | 425 |  |
| 5 | 76A | VAL | 3.55 | 12 | 714 |  |
| 6 | 80A | ALA | 3.56 | 14 | 749 |  |
| 1 | 30A | PHE | 3.72 | 25 | 239 | IL10_11431898  (Herkinorin) |
| 2 | 30A | PHE | 3.29 | 14 | 244 |  |
| 3 | 30A | PHE | 3.67 | 27 | 238 |  |
| 4 | 30A | PHE | 3.69 | 11 | 242 |  |
| 5 | 37A | PHE | 3.45 | 3 | 317 |  |
| 6 | 37A | PHE | 3.62 | 4 | 318 |  |
| 7 | 38A | GLN | 3.68 | 19 | 330 |  |
| 8 | 76A | VAL | 3.72 | 6 | 704 |  |
| 9 | 94A | LEU | 3.72 | 28 | 873 |  |
| 10 | 94A | LEU | 3.89 | 8 | 874 |  |
| 1 | 26A | LEU | 3.69 | 29 | 199 | IL10_45273151  (mulberrofuran W) |
| 2 | 26A | LEU | 3.46 | 26 | 200 |  |
| 3 | 30A | PHE | 3.67 | 28 | 238 |  |
| 4 | 30A | PHE | 3.6 | 18 | 242 |  |
| 5 | 30A | PHE | 3.93 | 16 | 244 |  |
| 6 | 33A | VAL | 3.6 | 8 | 277 |  |
| 7 | 37A | PHE | 3.76 | 2 | 317 |  |
| 8 | 37A | PHE | 3.95 | 13 | 318 |  |
| 9 | 72A | TYR | 3.52 | 21 | 664 |  |
| 10 | 72A | TYR | 3.98 | 22 | 662 |  |
| 11 | 72A | TYR | 3.56 | 23 | 663 |  |
| 12 | 80A | ALA | 3.51 | 10 | 739 |  |
| 13 | 94A | LEU | 3.52 | 26 | 873 |  |
| 14 | 98A | LEU | 3.6 | 24 | 909 |  |

Pi-stacking Interactions of top nine compounds.

| **Index** | **Residue** | **AA** | **Distance** | **Ligand Atoms** | **Protein-Ligand complex** |
| --- | --- | --- | --- | --- | --- |
| 1 | 53A | TYR | 4.85 | 3, 4, 5, 6, 7, 36 | IFNG_A_6000 |
| 1 | 95A | TRP | 4.18 | 12, 13, 14, 15, 16, 17 | IL4_13393486 |
| 2 | 95A | TRP | 3.71 | 12, 13, 14, 15, 16, 17 |  |
| 3 | 95A | TRP | 4.08 | 10, 11, 12, 17, 21 |  |
| 1 | 30A | PHE | 4.63 | 17, 18, 19, 20, 21, 32 | IL10_9851833 |
| 1 | 30A | PHE | 5.02 | 16, 17, 18, 19, 32 | IL10_11431898 |
| 1 | 37A | PHE | 3.8 | 7, 8, 9, 10, 11, 12 | IL10_45273151 |

Hydrogen Bond Interactions of top nine compounds.

| **AA** | **Distance H-A** | **Distance D-A** | **Donor Angle** | **Protein Donor** | **Side Chain** | **Donor Atom** | **Acceptor Atom** | **Protein-Ligand Complex** |
| --- | --- | --- | --- | --- | --- | --- | --- | --- |
| LYS | 2.21 | 3.21 | 173.13 | Yes | Yes | 175 [N3+] | 43 [O3 | IFNG_A_99620 |
| LYS | 3.22 | 4.09 | 146.63 | Yes | Yes | 166 [N3+] | 31 [O3] | IFNG_A_44568920 |
| ASP | 2.25 | 2.93 | 124.9 | No | Yes | 33 [O.CO_2_] | 825 [O-] | IFNG_A_44568920 |
| CYS | 2.34 | 2.7 | 101.15 | No | No | 22 [O3] | 497 [O2] | IL4_13393486 |
| LYS | 2.59 | 3.01 | 106.41 | No | No | 20 [O3] | 908 [O2] |  |

Pi-cation Interactions of top nine compounds.

| **Index** | **Residue** | **AA** | **Distance** | **Offset** | **Protein Charged** | **Ligand Group** | **Ligand Atoms** | **Protein-Ligand Complex** |
| --- | --- | --- | --- | --- | --- | --- | --- | --- |
| 1 | 57A | ARG | 4.42 | 1.23 | Yes | Aromatic | 10, 11, 12, 17, 21 | IL4_13393486 |
