# Supplementary material for: Interleukin-10 as Covid-19 biomarker targeting KSK and its analogues: Integrated network pharmacology
Source: PLoS One. 2023 Mar 29;18(3):e0282263. doi: 10.1371/journal.pone.0282263 (PMC10057793; doi:10.1371/journal.pone.0282263)
Supplement: S6 File — (DOCX) [file pone.0282263.s006.docx]

Summary Statistics of NetworkAnalyzer.

| Summary Statistics – NetworkAnalyzer | |
| --- | --- |
| Number of nodes | 29 |
| Number of edges | 132 |
| Avg. number of neighbors | 9.034 |
| Network diameter | 3 |
| Network radius | 2 |
| Characteristic path length | 1.793 |
| Clustering coefficient | 0.573 |
| Network density | 0.323 |
| Network heterogeneity | 0.779 |
| Network centralization | 0.459 |
| Connected components | 1 |
